# Supplementary material for: Discovery of Late Intermediates in Methylenomycin Biosynthesis Active against Drug-Resistant Gram-Positive Bacterial Pathogens
Source: J Am Chem Soc. 2025 Oct 27;147(44):40554–61. doi: 10.1021/jacs.5c12501 (PMC12593393; doi:10.1021/jacs.5c12501)
Supplement: Supplementary file 1 [file ja5c12501_si_001.pdf]

Supplementary Information

Discovery of late intermediates in methylenomycin biosynthesis active against drug-resistant Gram-positive bacterial pathogens

Christophe Corre,[†,‡,⁑] Gideon A. Idowu,[†,⁑,#] Lijiang Song,[†] Melanie E. Whitehead,[†] Lona M. Alkhalaf,[†,*] and Gregory L. Challis [†,§,¥,*]

[†]Department of Chemistry, University of Warwick, Coventry CV4 7AL, UK
[‡]School of Life Sciences, University of Warwick, Coventry CV4 7AL, UK
[§]Department of Biochemistry and Molecular Biology, Biomedicine Discovery Institute, Monash University, Clayton, Victoria 3800, Australia
[¥]ARC Centre of Excellence for Innovations in Peptide and Protein Science, Monash University, Clayton Victoria 3800, Australia

[#]Current addresses: Department of Chemistry, Federal University of Technology, PMB 704 Akure, Nigeria (G.A.I.); Nottingham University Hos-pitals; Queens Medical Centre, Derby Road, Nottingham, NG7 2UH, U.K (M.W.).

[⁑]Author contributions: C.C. and G.A.I. contributed equally.

Experimental procedures

Culture conditions for production of methylenomycins. The *Streptomyces* strains were grown on a modified SMMS agar medium,[1] containing 5mM each of NaH_2PO_4 and K_2HPO_4. After incubation at 30 °C for 72 h, the plates were frozen overnight. After defrosting, the agar and mycelia were filtered with cotton wool by centrifugation for 5 mins at 2000 rpm. The resulting aqueous sample was analysed by LC-MS or used to purify methylenomycin related metabolites.

Analytical methods. LC-MS analyses were carried out using a reverse phase column (Agilent C18, 150 x 4.6 mm, 5 µm) connected to an Agilent 1100 HPLC instrument. The outflow was routed to a Bruker (MaXis™ Impact) High Resolution-Mass Spectrometer (HR-MS) fitted with an electrospray ionisation (ESI) source operating in positive or negative mode.

Methylenomycin related metabolites were separated using the following HPLC procedure from 20 uL of crude extract. Solvent A = H_2O (0.1 % formic acid) Solvent B = MeOH (0.1 % formic acid). A:B 5:25 (0-5 min), 5:25 to 63:37 (5-21 min), 63:37 -45:55 (21-30 min), 45:55 -0:100 (30-35 min), 0:100 (35-40 min), 0:100 -75:25 (40-45 min), 75:25 (45-55 min).

Purification and characterisation of pre-methylenomycin C lactone (5)

SMMS agar (16 x 50 mL), was inoculated with spores of *S. coelicolor* W86. After incubation for 5 days at 30 ºC, cultures were combined, extracted with ethyl acetate (800 mL) and dried over $MgSO_4$. The solvent was removed *in vacuo* and the residue redissolved in water/methanol (1:1, 2 mL). Pre-MmCl was purified by preparative HPLC. Solvent A = H_2O (0.1 % formic acid) Solvent B = MeOH (0.1 % formic acid). A:B 95:5 (0-5 min), 95:5 to 0:100 (5-25 min). Flow rate 20 $mLmin^{-1}$

The collected fractions were combined, CH_3OH was removed *in vacuo* and the remaining aqueous fraction was extracted with ethyl acetate (2 x 50 mL). The ethyl acetate was dried over $MgSO_4$, filtered and removed *in vacuo*. NMR spectroscopy was conducted on a Bruker AV700 spectrometer equipped with a TCI cryoprobe.

δ_H (700 MHz, $CDCl_3$), 1.73 (3H, s, C8), 2.25 (3H, s, C9), 3.29 (1H, dt, J 7.30, 3.30, H2), 3.63 (1H, d, J 7.30, H1), 4.40 (1H, dd, J 3.30, 9.70, H7b), 4.52 (1H, t, J 9.70, H7a) **δ_C** (175 MHz, $CDCl_3$), 8.3 (C8), 15.5 (C9), 45.1 (C2), 48.8 (C1), 68.3 (C7), 137.3 (C5), 165.6 (C4), 173.3 (C6), 206.8 (C3), HR-ESI-MS *m/z* 167.0701 $[M + H]^+$ (Calculated for $[C_9H_{11}O_3]^+$: 167.0708).

Conversion of pre-methylenomycin C lactone (5) to pre-methylenomycin C (6)

5 (1 mg) was resuspended in THF (250 µL) and sodium hydroxide (10 mM, 250 µL) was added. The mixture was stirred at room temperature for 6 hours. The product was dried *in vacuo*

δ_H (700 MHz, D_2O), 1.71 (3H, s, H8), 2.09 (3H, s, H9), 2.88 (1H, q, J 6.70, 5.90, H2), 3.76 (1H, d, J 6.70, H1), 3.79 (1H, dd, J 5.90, 12.70, H7b), 3.89 (1H, dd, J 12.70, H7a), **δ_C** (176 MHz, D_2O), 7.2 (C8), 15.3 (C9), 50.3 (C2), 56.5 (C1), 59.9 (C7), 136.8 (C5), 173.2 (C4), 213.0 (C3), signal for C6 was not observed due to low intensity; HRMS Calculated for $[C_9H_{13}O_4]^+$: 185.0814, observed: 185.0807.

Purification and characterisation of methylenomycin D1 (7) and methylenomycin D2 (8)

SMMS agar (12 x 50 mL) was inoculated with spores of *S. coelicolor* W108 and incubated for 7 days at 30 ºC. The plates were combined, acidified to pH 3 and extracted with 700ml of ethyl acetate. The ethyl acetate was dried over magnesium sulphate and removed *in vacuo*. MmD1 (**7**) and MmD2 (**8**) were first copurified by silica chromatography (toluene-acetic acid 9:1). The mixture of diastereoisomers was then separated by preparative HPLC using the elution conditions described for **5**, except CH_3CN was used in place of CH_3OH to give **9** (2 mg) and **10** (4mg).

Methylenomycin D1 (**7**): ^1H NMR (700 MHz, $CDCl_3$): δ 1.20 (3H, d, 3H's on C7, J = 7.5 Hz), 1.76 (3H, s, 3H's on C8), 2.07 (3H, s, 3H's on C9), 2.72 (1H, m, H2), 3.77 (1H, d, H1, J = 7.3 Hz) ppm. ^{13}C NMR (175 MHz, $CDCl_3$): δ 8.3 (C8), 11.9 (C7), 15.8 (C9), 41.9 (C2), 53.2 (C1), 137.8 (C5), 163.3 (C4), 208.5 (C3) ppm. HR-ESI-MS *m/z* = 169.0860 $[M + H]^+$ (Calculated $[C_9H_{13}O_3]^+$: 169.0860).

Methylenomycin D2 (**8**): ^1H NMR (700 MHz, $CDCl_3$): δ 1.27 (3H, d, 3H's on C7, J = 7.4 Hz), 1.75 (3H, s, 3H's on C8), 2.09 (3H, s, 3H's on C9), 2.65 (1H, m, H2), 3.21 (1H, s, H1) ppm. ^{13}C NMR (175 MHz, $CDCl_3$): δ 8.4 (C8), 15.5 (C9), 15.6 (C7), 44.3 (C2), 56.2 (C1), 137.3 (C5), 162.6 (C4), 208.5 (C3) ppm. HR-ESI-MS *m/z* = 169.0861 $[M + H]^+$ (Calculated $[C_9H_{13}O_3]^+$: 169.0860).

Purification of methylenomycin C (2)

Production of **2** was conducted as described for **7** and **8**, but incubation was only for 36 hours. **2** was purified from the acidified culture extract by silica chromatography (toluene-acetic acid 9:10 ^{1}H and ^{13}C NMR data obtained were consistent with those determined previously for **2**.[2]

MIC and MBC determinations

MICs were determined by broth microdilution in 96-well microtiter plates according to the CLSI guidelines.[3, 4] Cells growing in exponential phase were diluted to *ca.* 10^5 CFU/ml into cation-adjusted Mueller-Hinton broth (MHB) before the addition of methylenomycin compounds in increasing concentrations. *Enterococci* strains were grown and diluted with Medium 92 (Trypticase soy broth 30 g, yeast extract 3 g, distilled water up to 1L). Strains were incubated for 20 h (or up to 48 h in the case of *Streptomyces* spp. and yeast) before visual inspection for growth.

Minimum bactericidal concentrations (MBCs) were determined by sub-culturing wells from MIC assays onto antibiotic-free agar plates and incubating overnight at 36 °C or at 30 °C for 4 days in the case of *Streptomyces* spp. The MBCs were further confirmed by resazurin-reduction assay with AlamarBlue™ dye as described previously.[5,6] Sequential passage of *E. faecium* 64/3 through pre-methylenomycin C lactone (**5**) and vancomycin was carried out as described by Ling *et al.*[4]

Methods for construction of modified cosmids, plasmids, and strains

Construction of plasmids pCC003 - pCC015 and introduction into* S. coelicolor *M145 to generate* S. coelicolor *W89, W95, W86, W100 and W108 respectively

Putative biosynthetic genes were inactivated on the cosmid C73-787, which contains the entire methylenomycin biosynthetic gene cluster as well as an integrative cassette, via in-frame scar deletions and *mmyR* was replaced by an apramycin resistance cassette using PCR-targeting methodology (Figure S1).[7] The cosmid was derived from the *S. coelicolor* SCP1 ordered cosmid library.[8] The apramycin resistance cassette was amplified from pW60[9] using the forward primers A, B, C, D, and E and the reverse primers A′, B′, C′, D′ and E′ (Table S2) for *mmyR*, *mmyD*, *mmyE*, *mmyF* and *mmyO* replacement, respectively. After the separate replacement of *mmyD*, *mmyE*, *mmyF* and *mmyO* with the resistance

cassette gene, the *aac(3)-IV* gene was removed using the flip recombinase,[7] leaving an in-frame scar deletion between the start and stop codon of each gene. The same methodology was used to replace the transcriptional repressor, *mmyR*, with the apramycin resistance cassette in each resulting construct. The resulting cosmids lacking *mmyR* and with deletions in *mmyD*, *mmyE*, *mmyF,* or *mmyO* were named pCC012, pCC013, pCC014, and pCC015, respectively. The construct with only *mmyR* replaced by the apramycin resistance cassette was named pCC003. The desired deletion of genes on cosmid C73-787 was confirmed by PCR using primer pairs F/ F′, G/G′, H/H′, I/I′ and J/J′ (Table S3), which are complementary to regions upstream and downstream of *mmyR*, *mmyD*, *mmyE*, *mmyF* and *mmyO*, respectively. The primers K/K′, L/L′, M/M′ and N/N′ (Table S4) were used to sub-clone *mmyD*, *mmyE*, *mmyF* and *mmyO* genes into pOSV556 to generate pCC016, pCC017, pCC018 and pCC019, respectively.[9]

pCC003 and each of pCC012-pCC015 was then introduced separately into *S. coelicolor* M145 (a derivative of *S. coelicolor* A 3(2) lacking SCP1 and SCP2) *via* conjugation with *E. coli* ET12567/pUZ8002.[7,10] This generated *S. coelicolor* W89 (*mmyR::apr*), W95 (Δ*mmyD* and *mmyR*::*apr*), W86 (Δ*mmyE* and *mmyR*::*apr*), W108 (Δ*mmyF* and *mmyR*::*apr*) and W100 (Δ*mmyO* and *mmyR*::*apr*). The engineered strains (W95, W86, W108, W100) were genetically complemented by integration of pCC016, pCC017, pCC018 and pCC019 within their genomic DNA, respectively, to generate strains W118 (W95 + *mmyD*), W115 (W86 + *mmyE*), W109 (W108 + *mmyF*) and W113 (W100 + *mmyO*) by conjugation with *E. coli* ET12567/pUZ8002 carrying each plasmid.[7,10] These strains were analysed by PCR (figure S2 and S3) and Southern blot hybridization to confirm that genetic disruption and complementation were correctly introduced. The *S. coelicolor* strains and plasmids used are summarized in Table S5.

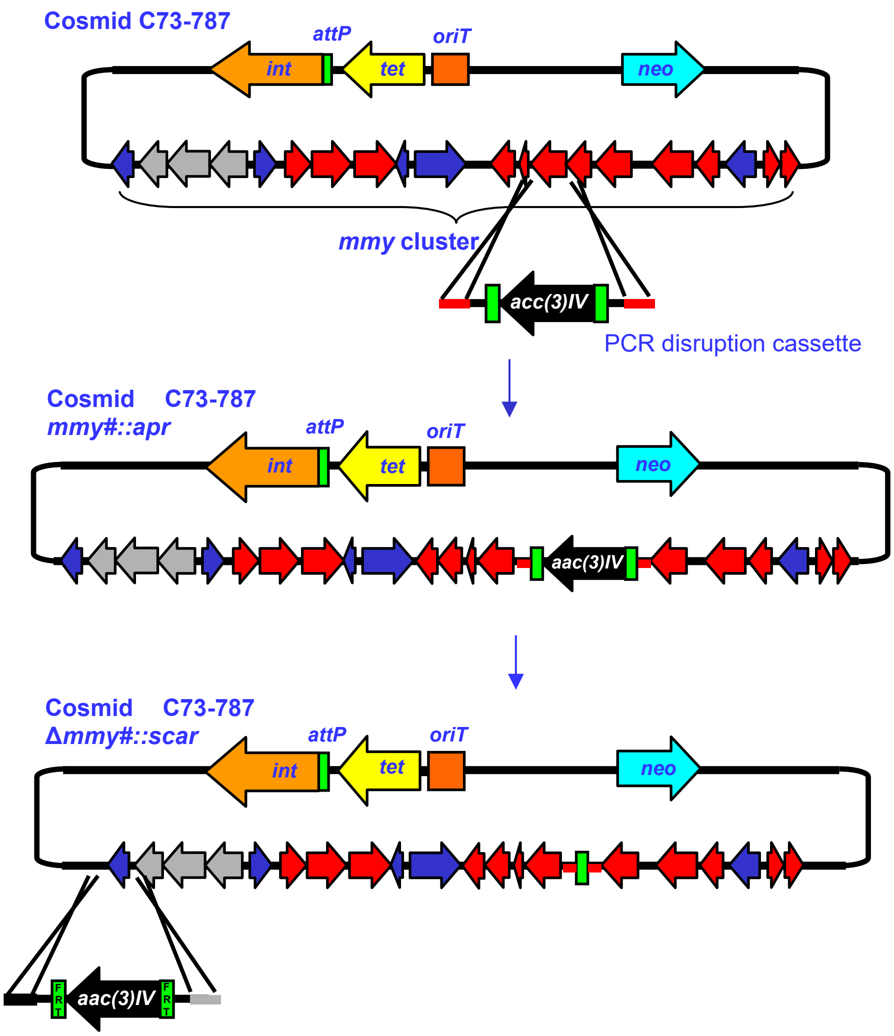

Figure S1. Schematic of PCR targeting method used to engineer cosmid C73-787. A PCR-generated disruption cassette containing the *aac(3)-IV* gene, which confers apramycin resistance, flanked by flip recombinase target (FRT) sites is inserted between the start and stop codons of the gene of interest (*mmy#*). The flip recombinase is then used to excise the *aac(3)-IV* gene, leaving an 81 bp in frame “scar” sequence between the start and stop codons. Finally, the *mmyR* gene is replaced with a PCR-generated disruption cassette containing the *aac(3)-IV* gene.

Table S1. Putative functions of proteins encoded by the methylenomycin biosynthetic gene cluster

Protein	Homologue (% identity)	Proposed function
MmyA	RedQ *Streptomyces coelicolor* (33)	Acyl carrier protein
MmyC	FabH *Escherichia coli* (39)	β-ketoacyl synthase III
MmyD	AvrD *Pseudomonas syringae* (28)	Butenolide synthase
MmyE	PlmM *Streptomyces* sp. HK803 (32)	Flavin-dependent enoyl reductase
MmyF	NtaB *Chelatobacter heintzii* (40)	Flavin reductase, partner to monooxygenase
MmyG	JadP *Streptomyces venezuelae* (29)	NAD(P)-dependent dehydrogenase
MmyK	Adk *Escherichia coli* (30)	Kinase
MmyO	LimB *Rhodococcus erythropolis* (43)	Flavin-dependent monooxygenase
MmyP	SsgB *Streptomyces griseus* (30)	Phosphatase
MmyQ	NdpG *Rhodococcus opacus* (30)	Coenzyme F-420-dependent reductase
MmyT	MtmZ *Streptomyces argillaceus* (31)	Thioesterase
MmyX	MmyK *Streptomyces coelicolor* (42)	Kinase
MmyY	JadX *Streptomyces venezuelae* (27)	Unknown – belongs to NTF2-like superfamily

Table S2. PCR primers used to create disruption cassettes for *mmyR, mmyD*, *mmyE*, *mmyF*, and *mmyO.*

Primers	Sequence
A	5’_CCCGTTTTCTCACGACCTTGAGAGGACTCGGGCGTTGTGTATTCCGGGGATCCGTCGACC_3’
A′	5'_CCGGAGCTCGTTGTCCGCCGGATCCTGGTCGCCGCAGTCATGTAGGCTGGAGCTGCTTC_3'
B	5’_ATCGATTGCACCTGTCGGGAAAAAACTGGAGGGTGCATGATTCCGGGGATCCGTCGACC_3’
B′	5'_CGTGTCCTGCAGGGCAGGCCGACGGTGGACAGTGGGTCATGTAGGCTGGAGCTGCTTC_3'
C	5’_GGCCCCCCACCGGGAACCAGTCATCCGAAGGGACAGATGATTCCGGGGATCCGTCGACC_3’
C′	5'_CGGACCCGGGCCGTGGTGTCACCGCCCTGCACGGCGTCATGTAGGCTGGAGCTGCTTC_3'
D	5’_GGCTGACTGTTTCCCCTTCTCCTCCAGGGAGTCCGCATGATTCCGGGGATCCGTCGACC_3’
D′	5'_CCTGAGCTCCATGGCGGACGAACTGCCGTCAGGTCCTCATGTAGGCTGGAGCTGCTTC_3'
E	5’_CCCACGCTGCAATTTCAGCGCGACCTTGAGCTGATAGAAATTCCGGGGATCCGTCGACC_3’
E′	5'_TCGCCGGGTGGAGCCGGTGAAGTGCGGGGCGACGTAGCGTGTAGGCTGGAGCTGCTTC_3'

Underlined sequences correspond to the 20 nucleotides P1 and 19 nucleotides P2 sequences described in the procedure developed by Gust *et al*.[7]

Table S3. PCR primers used to screen transconjugants for integration of each engineered cosmid into the chromosome of *S. coelicolor* M145

Primers	Sequence
F	5’_GCCATCGGTTGAATCCTG_3’
F′	5'_CAGGAAACGGACTGCCTG_3'
G	5’_CCGGCAATGACGAAATAG_3’
G′	5’_TGGCCAGGTTCATAGGAG_3’
H	5’_CACCGGGAACCAGTCATC_3’
H′	5'_GCTTGCCTCACCGAGTTG_3'
I	5'_CCCGCTGCCATGCGATTC_3'
I′	5’_ACCGCTACGGCCTAGTGC_3’
J	5’_GTGCACGGTTTACGGGATGAG_3’
J′	5’_ACGCCGATGCGTATCGGTTC_3’

Construction of plasmids pCC016 - pCC019 and pOSV556/mmyOF followed by introduction* into S. coelicolor *M145 to generate* S. coelicolor *W118, W115, W109 and W113, respectively.

The primers K/K’, L/L’, M/M’ and N/N’ (Table S4) were used to amplify the *mmyD*, *mmyE*, *mmyF* and *mmyO* genes and their native ribosome binding sites using C73-787 as a DNA template. Following digestion using pairs of restriction enzymes (i.e. *Hin*dIII/*Xho*I, *Ale*I/*Hin*dIII, *Bam*HI/*Hin*dIII and *Hin*dIII/*Xho*I, respectively), the *mmyD*, *mmyE*, *mmyF* and *mmyO* PCR products were isolated and subcloned in the integrative plasmid pOSV556 to generate pCC016, pCC017, pCC018 and pCC019 respectively. The plasmid pCC016-19 carrying a hygromycin resistance marker were then introduced into *S. coelicolor* M145 *via* conjugation from *E. coli* ET12567/pUZ8002 to generate *S. coelicolor* W118, W115, W109 and W113 (Table S5), respectively.

Table S4. PCR primers used to amplify *mmyD*, *mmyE*, *mmyF* and *mmyO* for cloning into pOSV556

Primers	Sequence
K	5’_GGGGGAAGCTTGAGAAGGGAGCGGACAT**ATG**CCAGTCAGCGGTTCCC_3’
K′	5'_GATAATCTCGAGGTGGACAGTGGG**TCA**AG_3'
L	5’_AATGCTCACGATTGTGAGGAGGGGCAG**ATG**CACG_3’
L′	5’_AATAATAAGCTTGCCCTGCACGGCG**TCA**G_3’
M	5’_AAAGGAAGCTTAGGAGGGTCCGC**ATG**GCTACG_3’
M′	5'_GGGAAACTCGAGCCGTCAGGTCC**TCA**TG_3'
M″	5'_AAACGCGGATCCAGGAGGGTCCGC**ATG**GCTACG_3’
N	5'_AAAGGGAAGCTTAGGAGGACGTTC**ATG**TACCCCG_3'
N′<br>N″O<br>O′	5’_GGGAAACTCGAGGGCGTGCACGGT**TTA**C_3’<br>5’_GGGAAAGGATCCGGCGTGCACGGT**TTA**C_3’<br>5’_ATATAGGATCCAGGAGGAACAGC**ATG**ACCACTG_3’<br>5’_ATTATTAAGCTTGGCAGTGTCCAGGAGCG_3’

Underlined sequences correspond to restriction sites used for cloning the inserts into pOSV556 (HindIII for K, L’, M, N, O’; XhoI for K’, M’, N’; AleI forL; BamHI for M’’, N’’, O); Start and stop codons are indicated in bold.

Table S5. List of plasmids and strains

Strains / Plasmids	Relevant properties	Reference
S. coelicolor strains		
M145	SCP1$^-$, SCP2$^-$ (methylenomycin non-producer)	10
W89	M145 containing pCC003::*attB* → *mmyR*::*apr*	This study
W95	M145 containing pCC012::*attB* → *mmyR*::*apr* and Δ*mmyD*	This study
W86	M145 containing pCC013::*attB* → *mmyR*::*apr* and Δ*mmyE*	This study
W108	M145 containing pCC014::*attB*→ *mmyR*::*apr* and Δ*mmyF*	This study
W100	M145 containing pCC015::*attB*→ *mmyR*::*apr* and Δ*mmyO*	This study
W118	W95 containing pCC016::*attB'*→ *mmyD* complementation	This study
W115	W86 containing pCC017::*attB'*→ *mmyE* complementation	This study
W109	W108 containing pCC018::*attB'*→ *mmyF* complementation	This study
W113	W100 containing pCC019::*attB'*→ *mmyO* complementation	This study
W110	M145 containing pOSV556/*mmyOF* integrated into *attB'*	This study
W301	W110 containg pIJ86/*mmr*	This study
W302	M145 with pIJ86/*mmr*	This study
Plasmids		
C73_787	Integrative cosmid containing the *mmy* gene cluster	8
pCC003	C73_787 with *mmyR*::*apr*	This study
pCC004	C73_787 with *mmyD::apr*	This study
pCC005	C73_787 with *mmyE::apr*	This study
pCC006	C73_787 with *mmyF*::*apr*	This study
pCC007	C73_787 with *mmyO::apr*	This study
pCC008	C73_787 with Δ*mmyD*	This study
pCC009	C73_787 with Δ*mmyE*	This study
pCC010	C73_787 with Δ*mmyF*	This study
pCC011	C73_787 with Δ*mmyO*	This study
pCC012	C73_787 with Δ*mmyD* and *mmyR*::*apr*	This study
pCC013	C73_787 with Δ*mmyE* and *mmyR*::*apr*	This study
pCC014	C73_787 with Δ*mmyF* and *mmyR*::*apr*	This study

pCC015	C73_787 with Δ*mmyO* and *mmyR*::*apr*	This study
pCC016	pOSV556t containing *mmyD* gene and artificial RBS	This study
pCC017	pOSV556t containing *mmyE* gene and artificial RBS	This study
pCC018	pOSV556t containing *mmyF* gene and artificial RBS	This study
pCC019	pOSV556t containing *mmyO* gene and artificial RBS	This study
pOSV556/ *mmyOF*	pOSV556 containing *mmyOF* under the control of the *ermE** promoter	This study
pIJ86/*mmr*	pIJ86 containing *mmr* under the control of the *ermE** promoter	This study

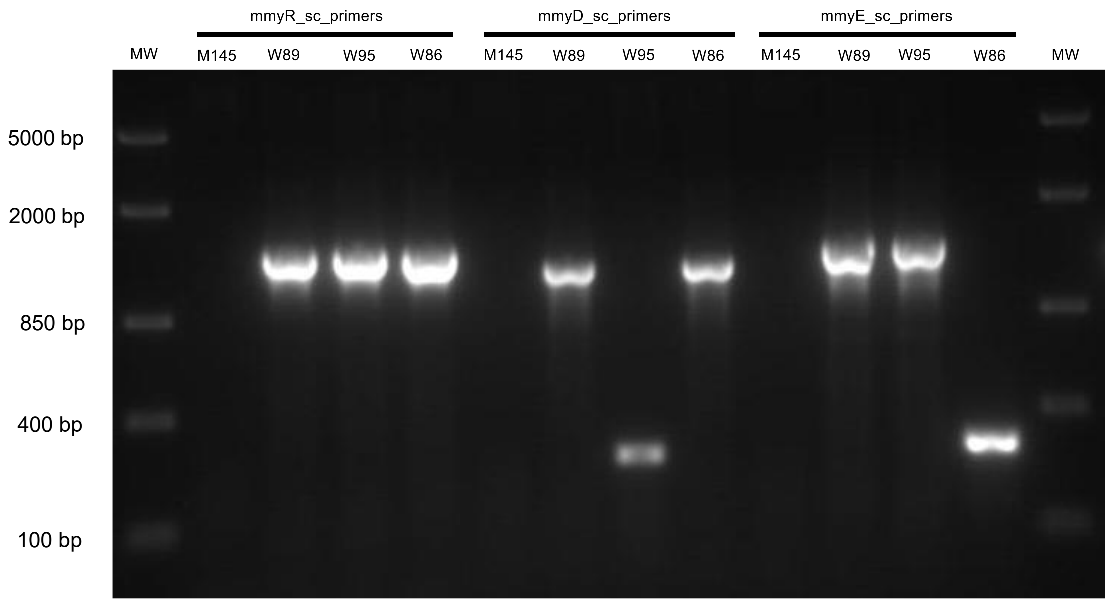

Figure S2. PCR analysis of genomic DNA from *S. coelicolor* M145, W89, W95 and W86.

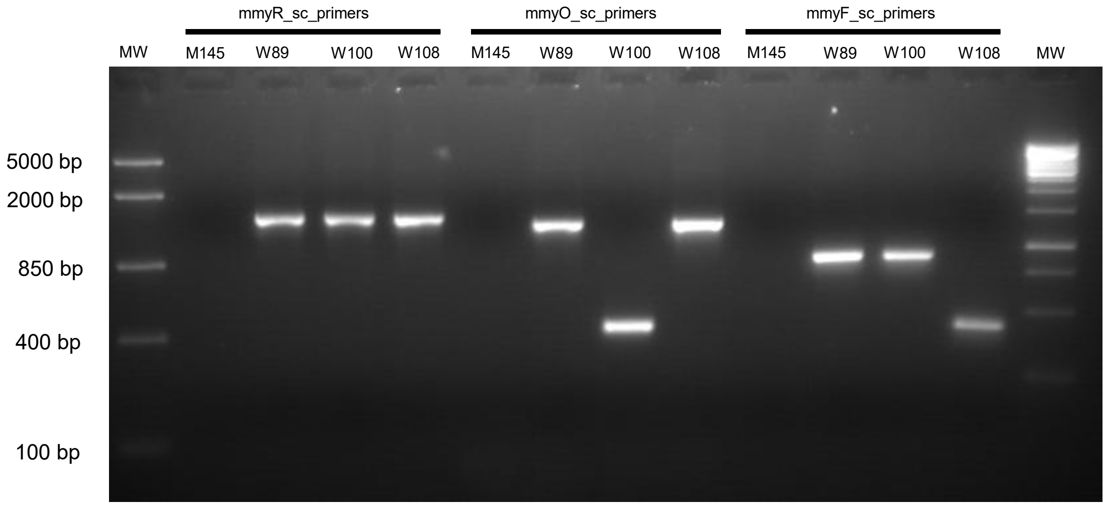

Figure S3. PCR analysis of genomic DNA from *S. coelicolor* M145, W89, W100 and W108

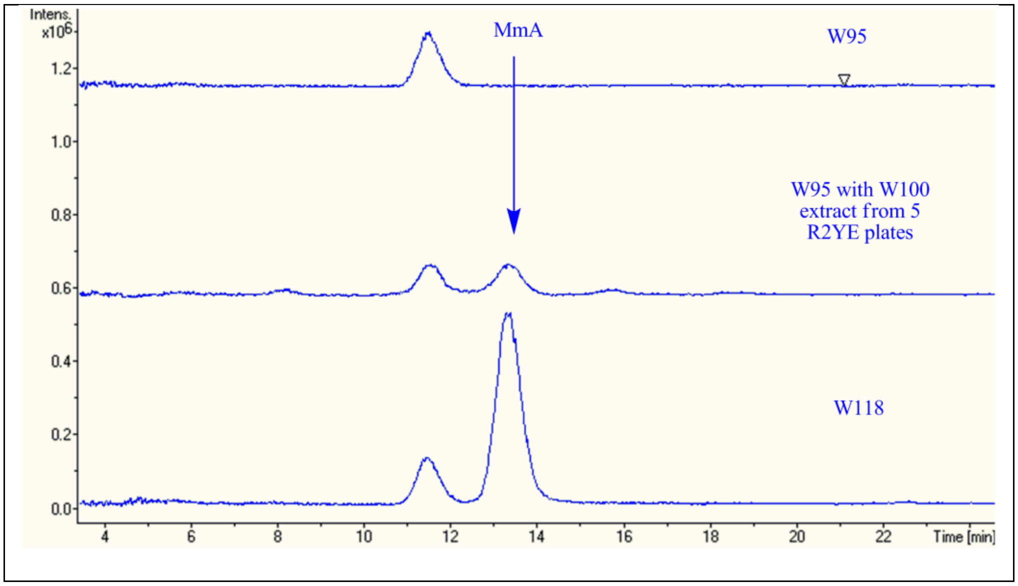

Figure S4. Genetic and chemical complementation of *S. coelicolor* W95 (Δ*mmyD*/*mmyR*::*apr*) restores methylenomycin A production. Extracted ion chromatogram at *m/z* = 183.0650 (corresponding to $[M+H]^+$ for **1**) from LC-MS analysis of extracts *S. coelicolor* W95 (top), *S. coelicolor* W95 fed with an organic extract of *S. coelicolor* W100 (middle), and *S. coelicolor* W118 (bottom).

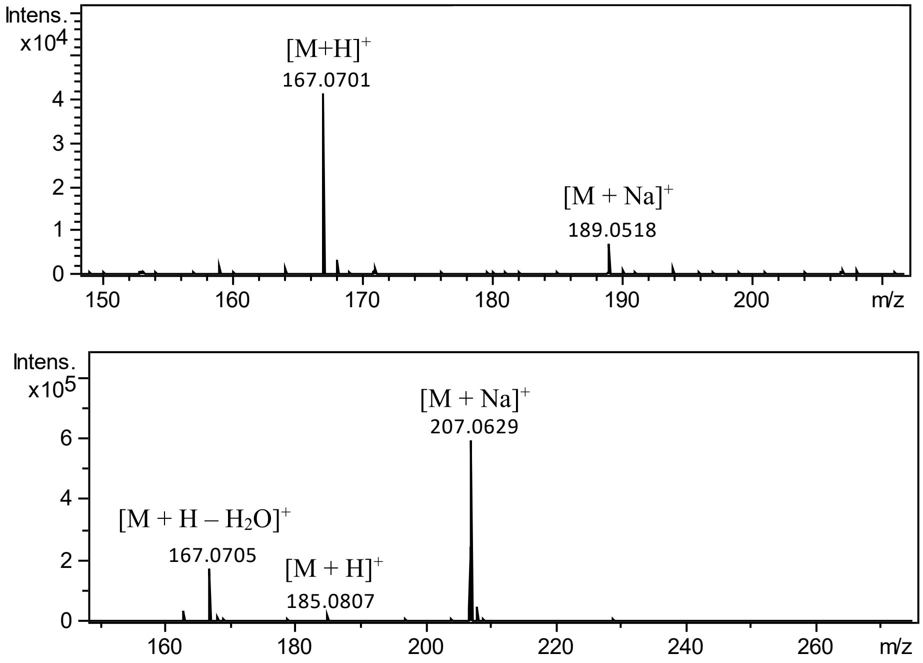

Figure S5. High-resolution mass spectra of pre-methylenomycin C lactone (**5**) (top) and pre-methylenomycin C (**6**) (bottom).

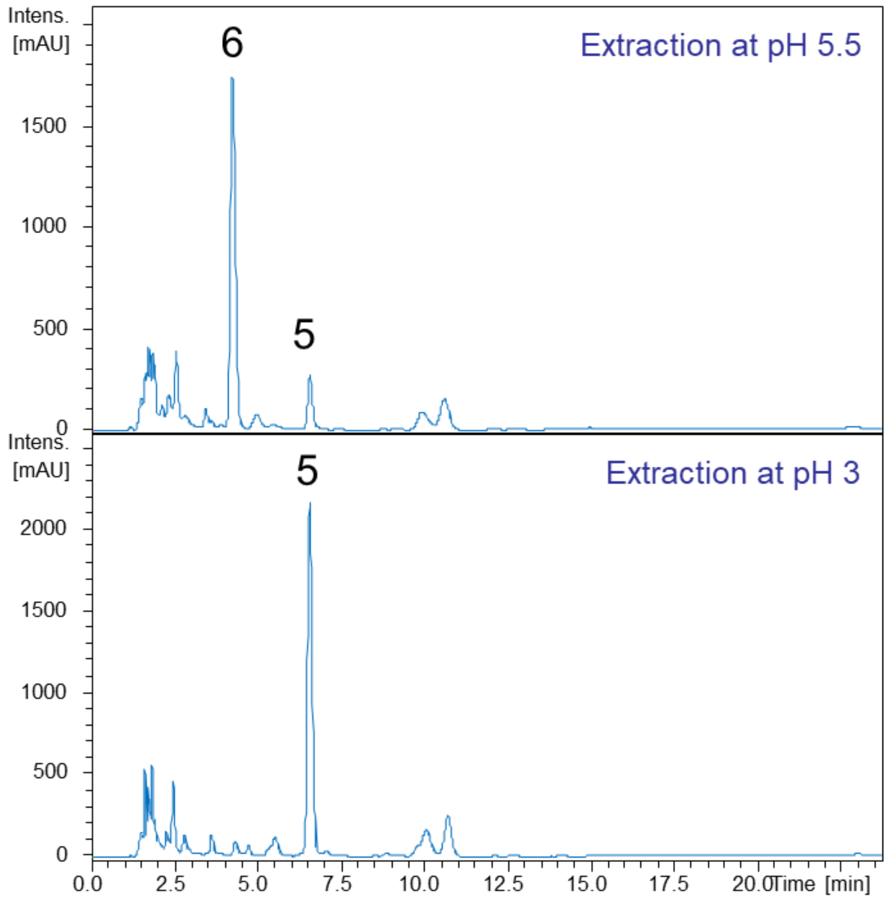

Figure S6 UV chromatograms at 230nm from HPLC purification of neutral (pH = 5.5; top) or acidic (pH = 3; bottom) extracts of *S. coelicolor* W86.

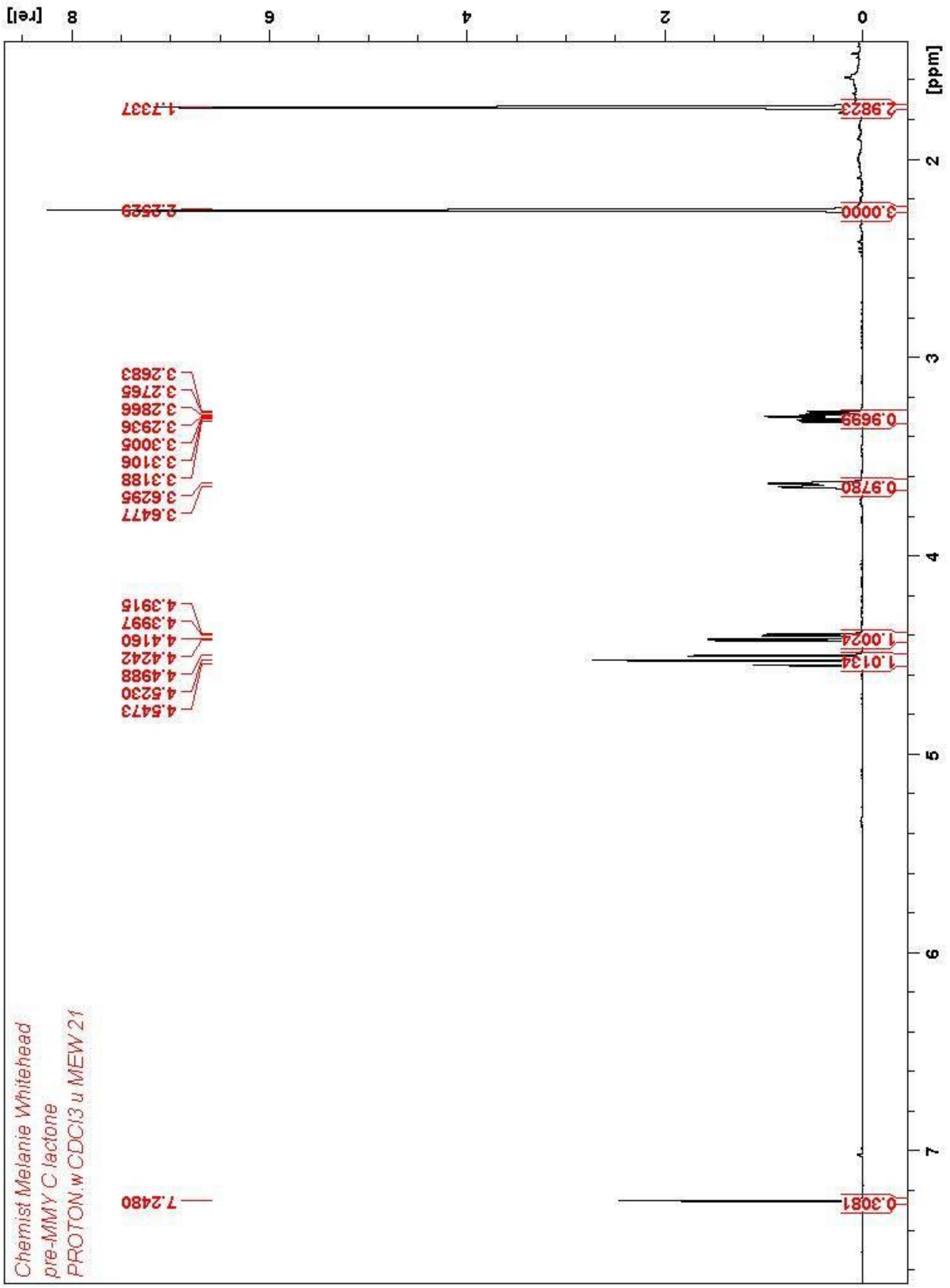

Figure S7. ^{1}H-NMR spectrum of pre-methylenomycin C lactone (**5**) in $CDCl_3$

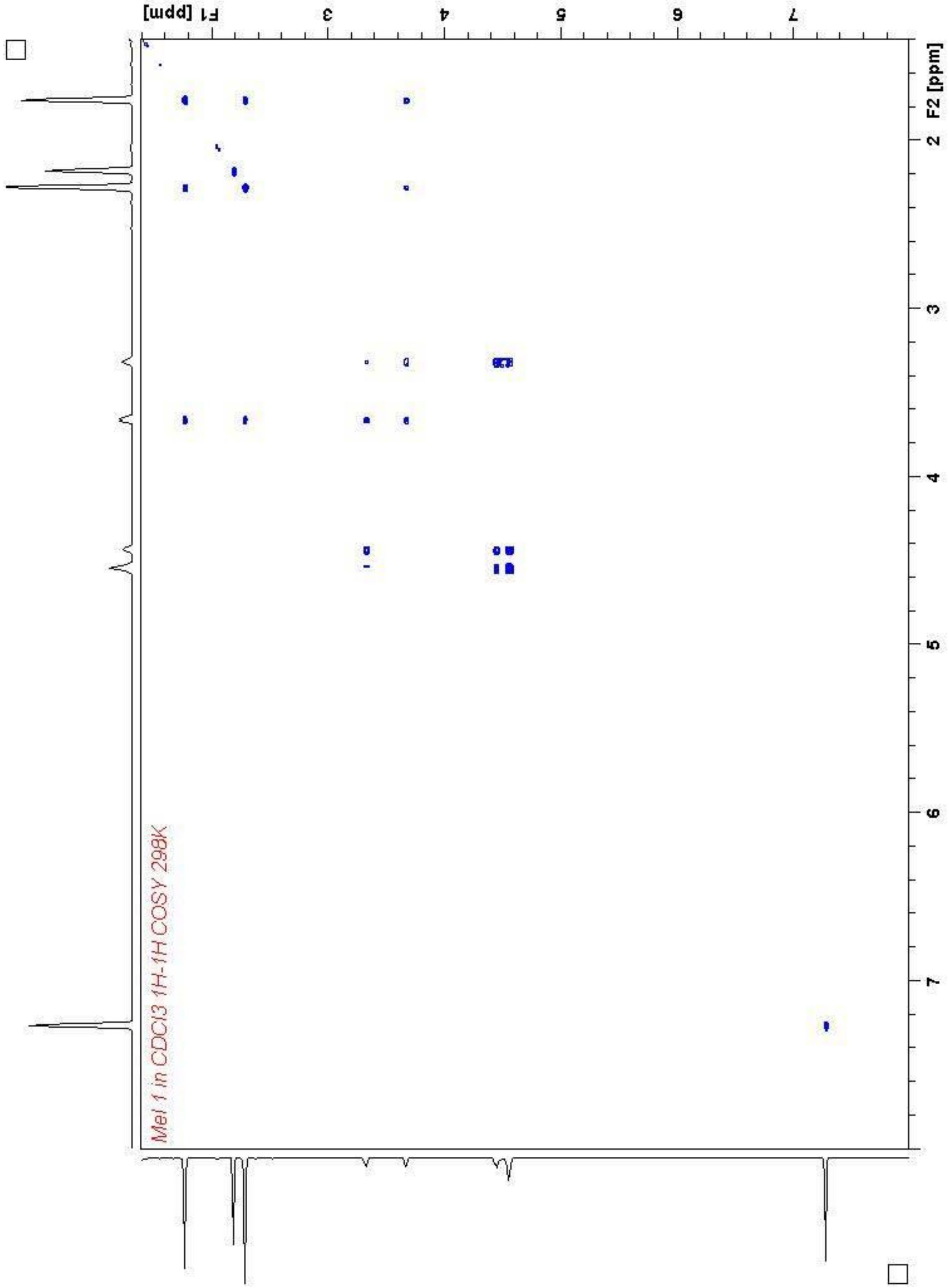

Figure S8. COSY spectrum of pre-methylenomycin C lactone (**5**) in $CDCl_3$

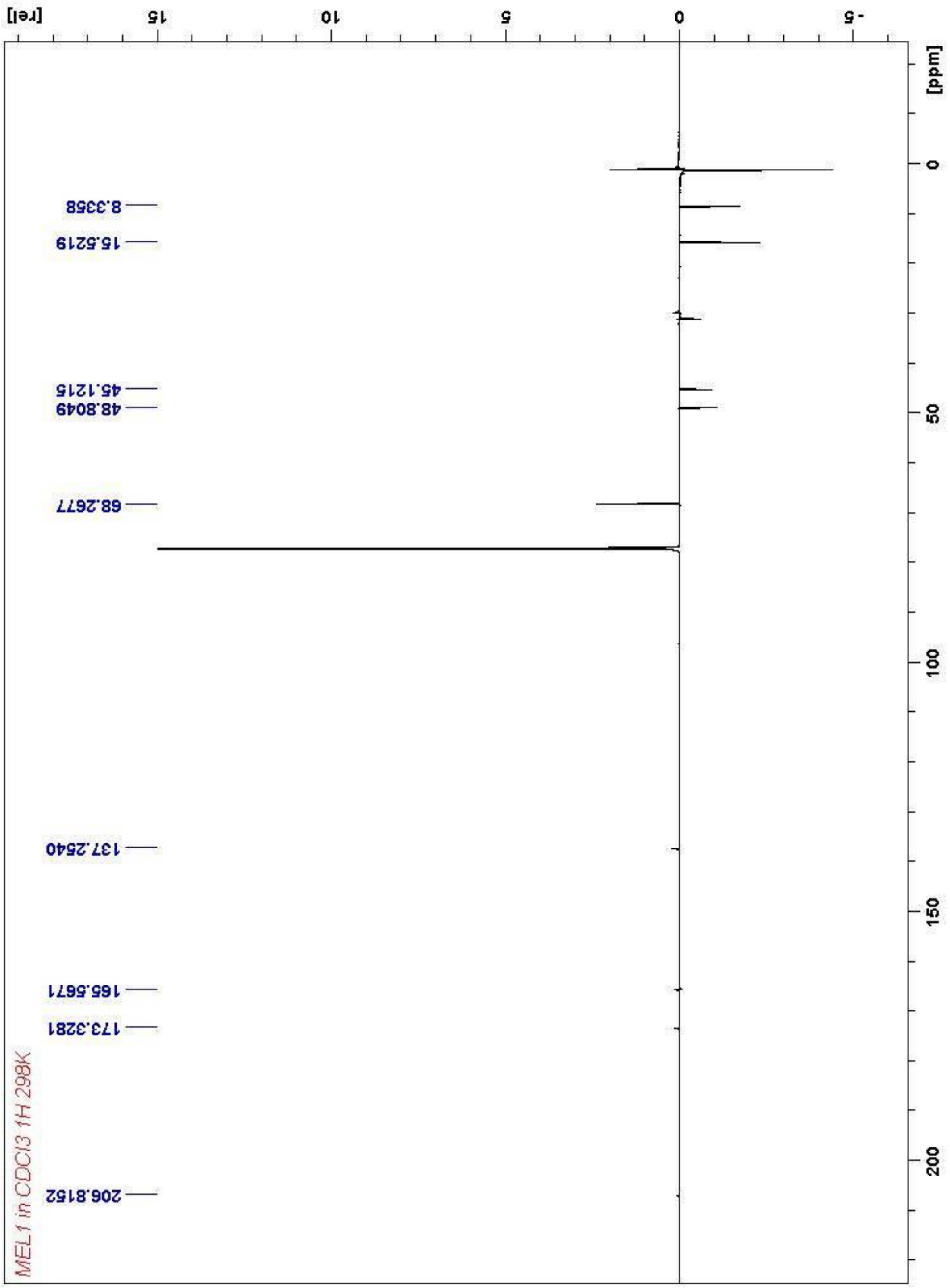

Figure S9. ^{13}C-NMR spectrum of pre-methylenomycin C lactone (**5**) in $CDCl_3$

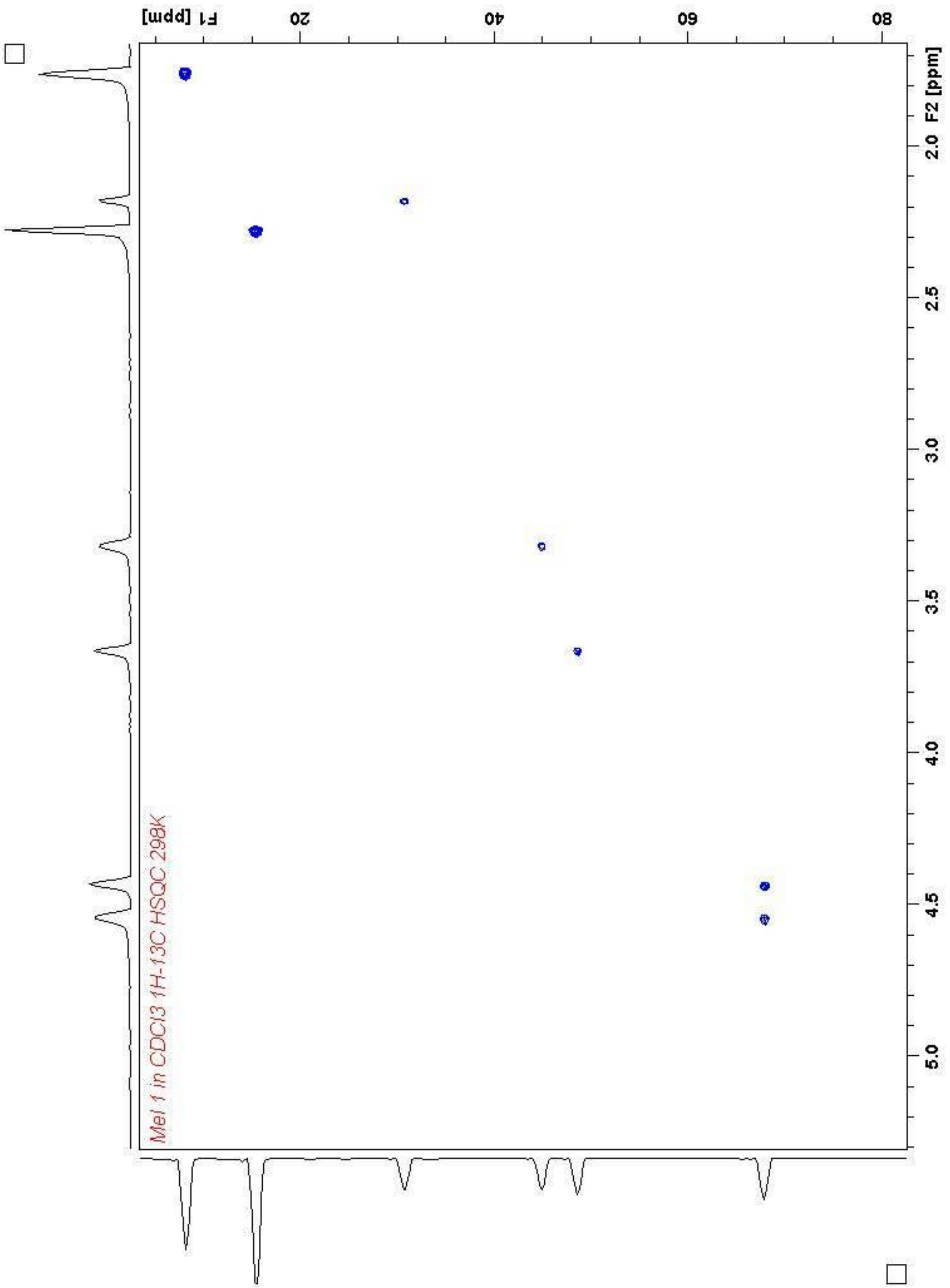

Figure S10. HSQC spectrum of pre-methylenomycin C lactone (**5**) in $CDCl_3$

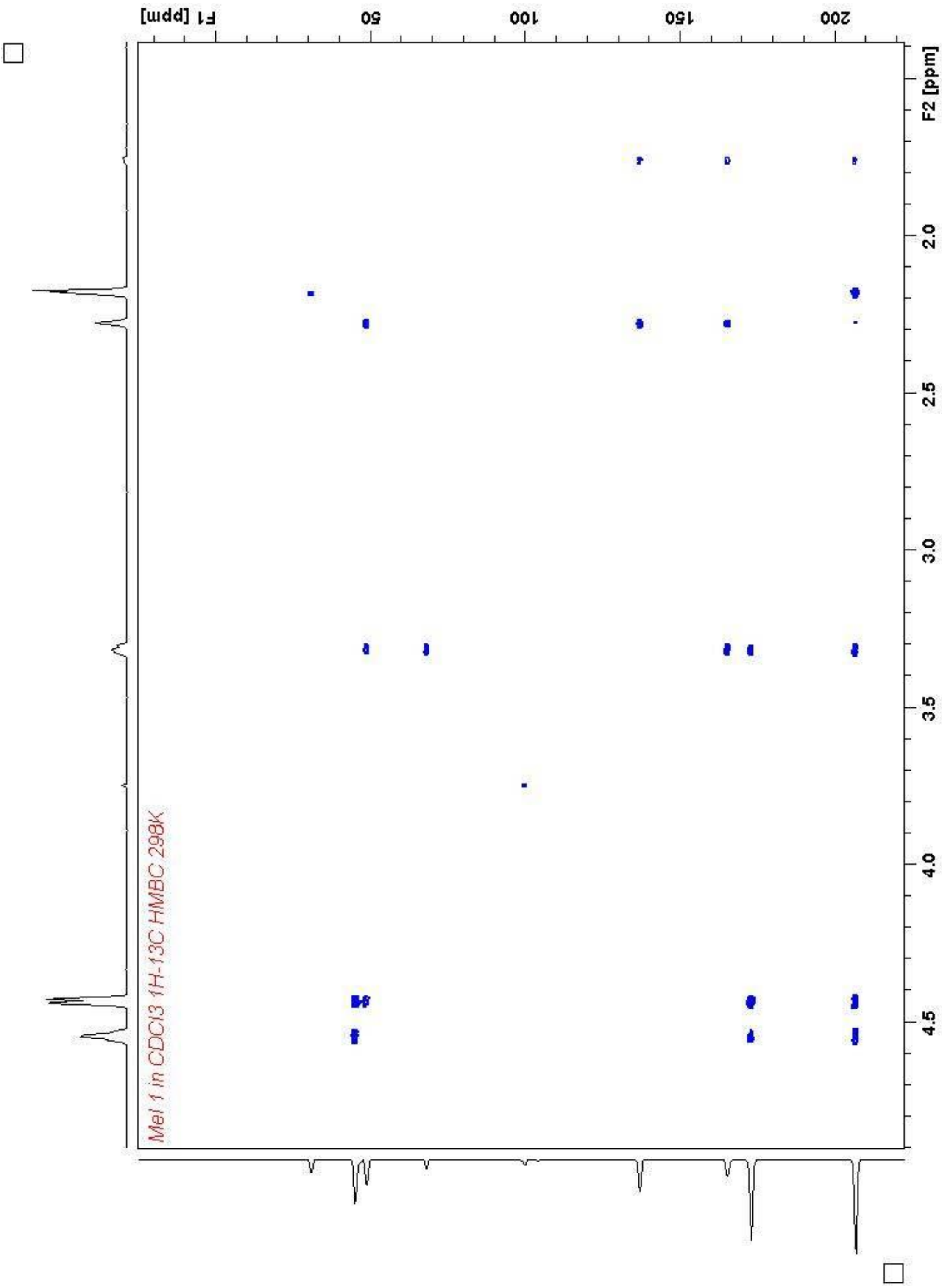

Figure S11. HMBC spectrum of pre-methylenomycin C lactone (**5**) in $CDCl_3$

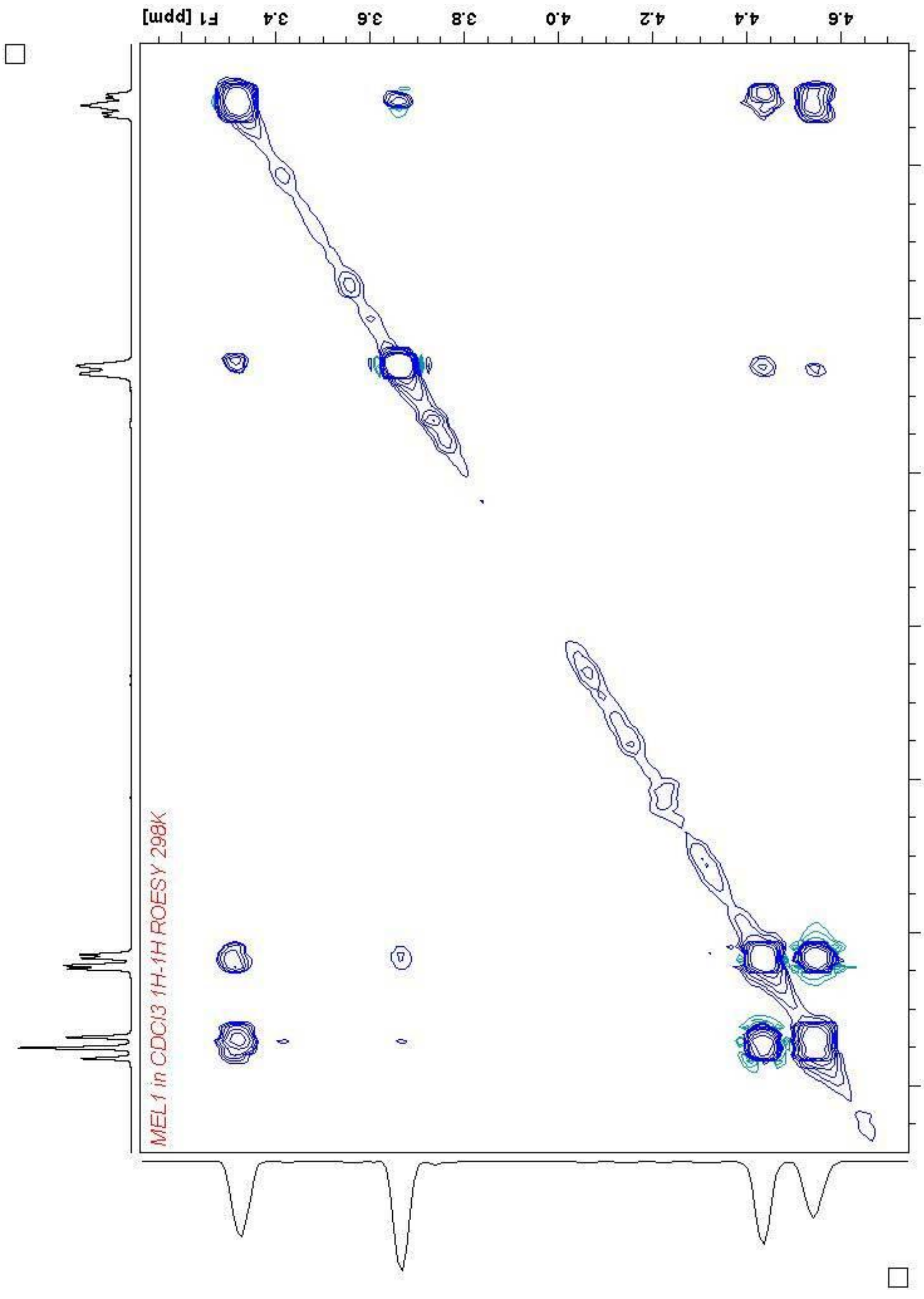

Figure S12. ROESY spectrum of pre-methylenomycin C lactone (**5**) in $CDCl_3$

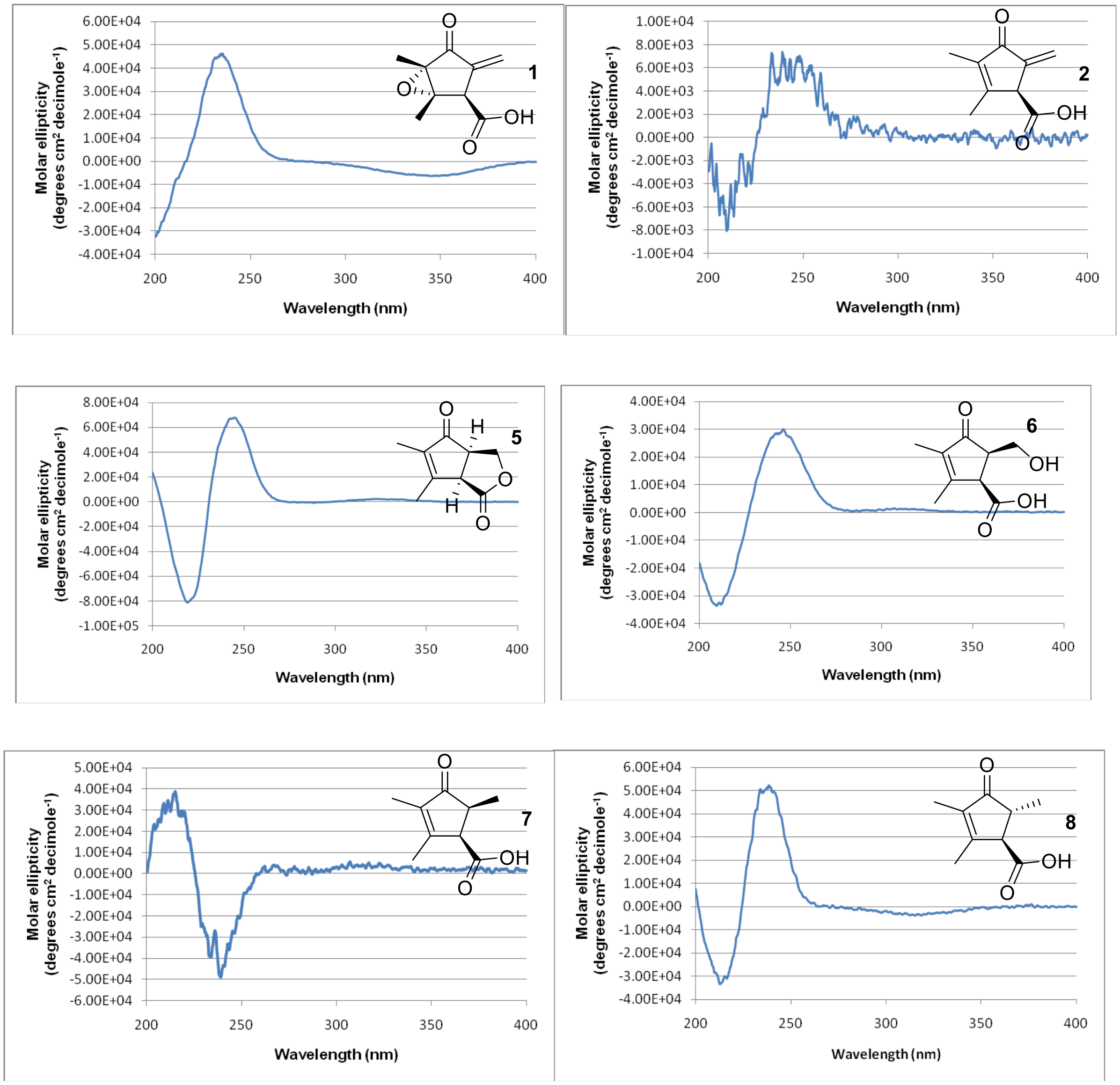

Figure S13. CD spectrum of methylenomycin A (**1**), methylenomycin C (**2**), pre-methylenomycin C lactone (**5**), pre-methylenomycin C (**6**), methylenomycin D1 (**7**) and methylenomycin D2 (**8**)

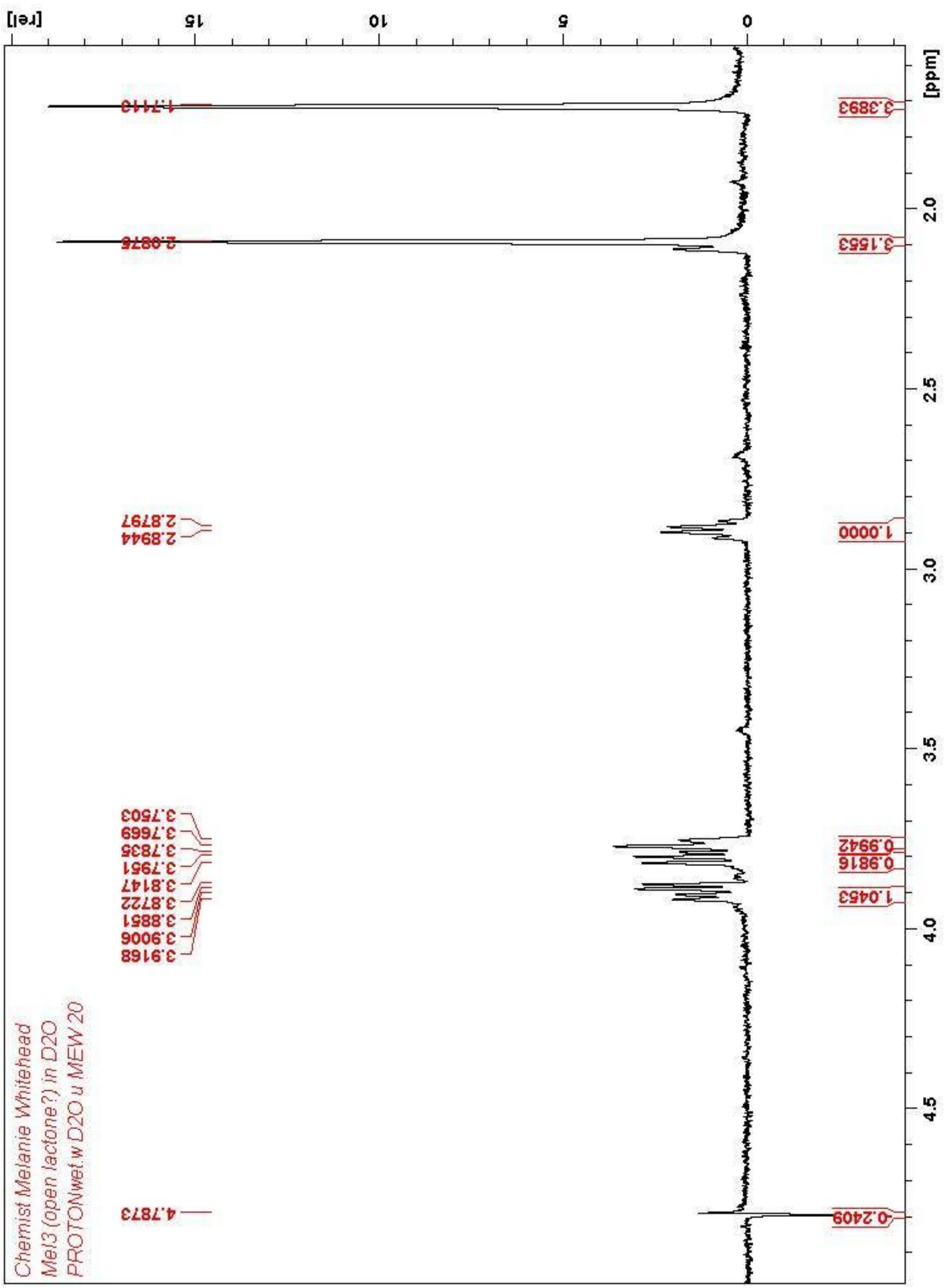

Figure S14. ^{1}H-NMR spectrum of pre-methylenomycin C (**6**) in D_2O

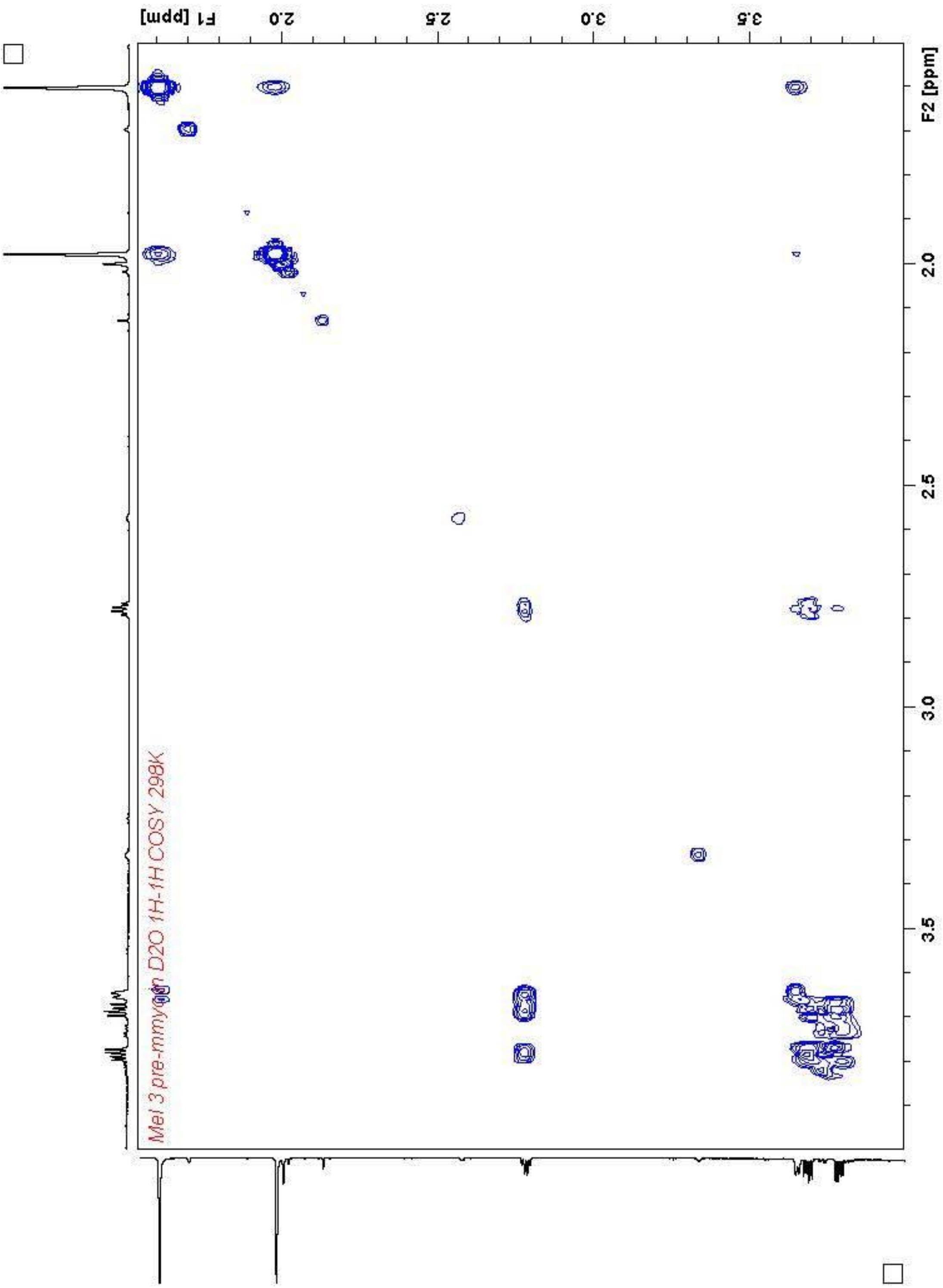

Figure S15. COSY spectrum of pre-methylenomycin C (**6**) in D_2O

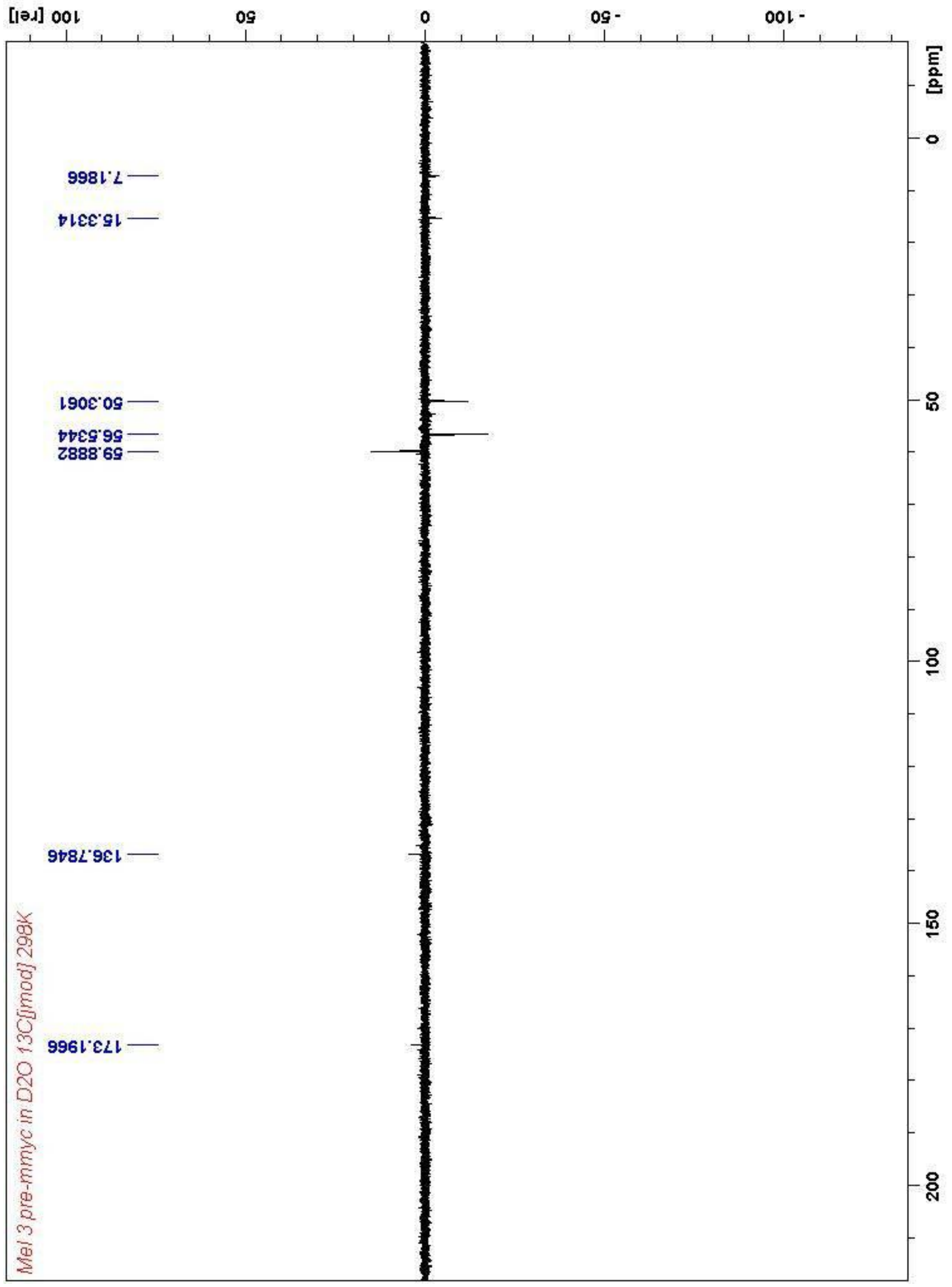

Figure S16. ^{13}C-NMR spectrum of pre-methylenomycin C (**6**) in D_2O

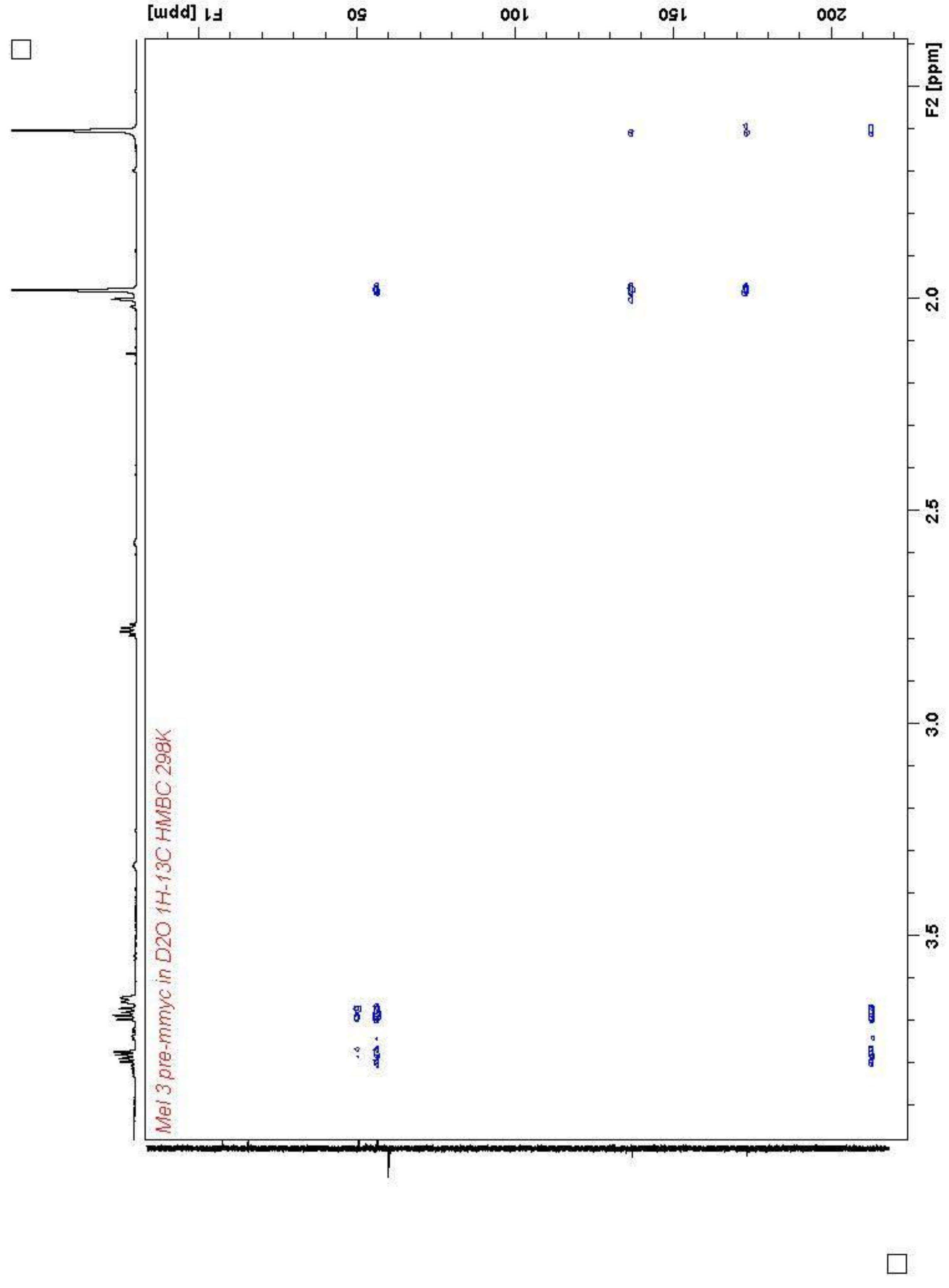

Figure S17. HMBC spectrum of pre-methylenomycin C (**6**) in D_2O

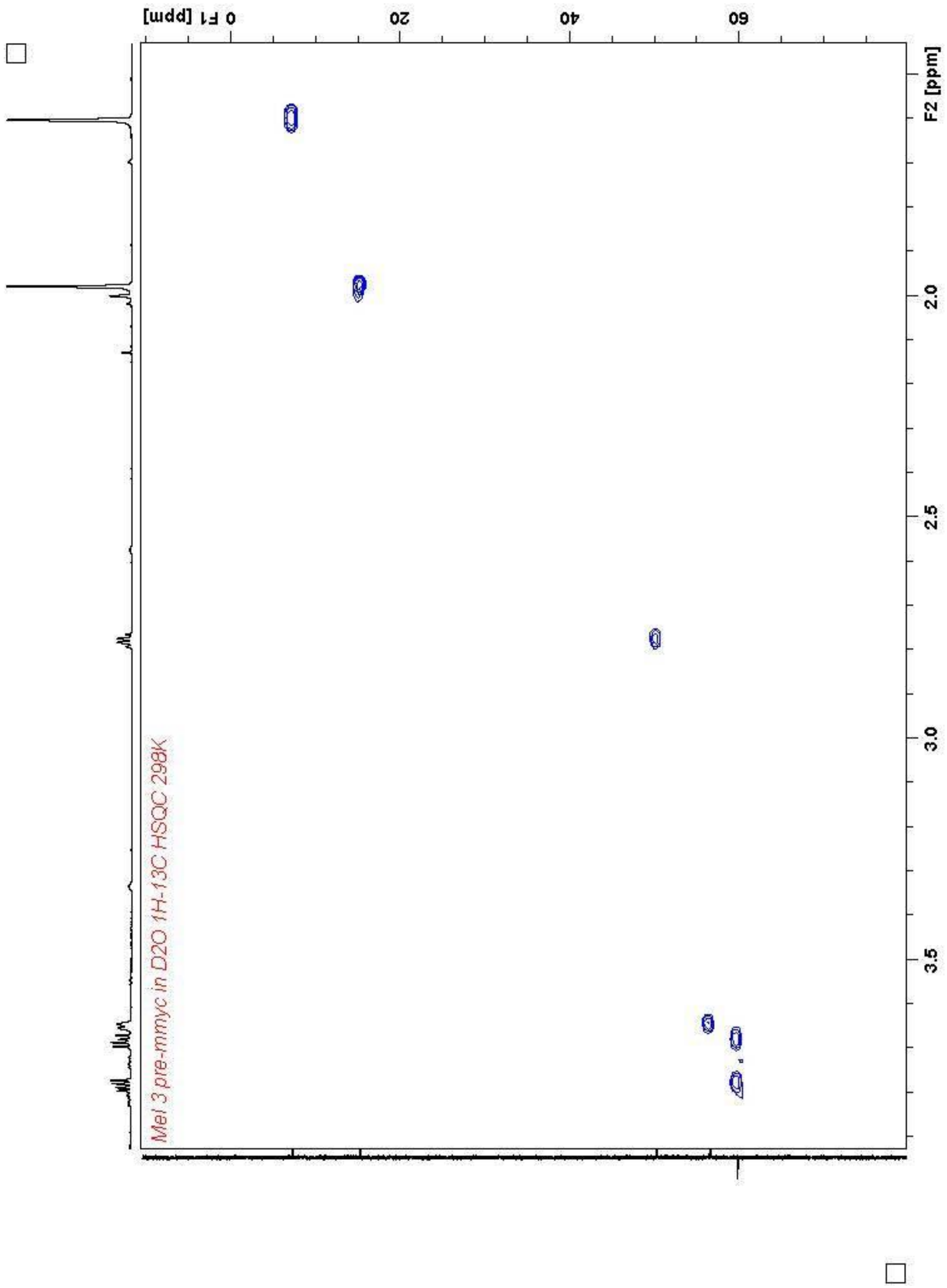

Figure S18. HSQC spectrum of pre-methylenomycin C (**6**) in D_2O

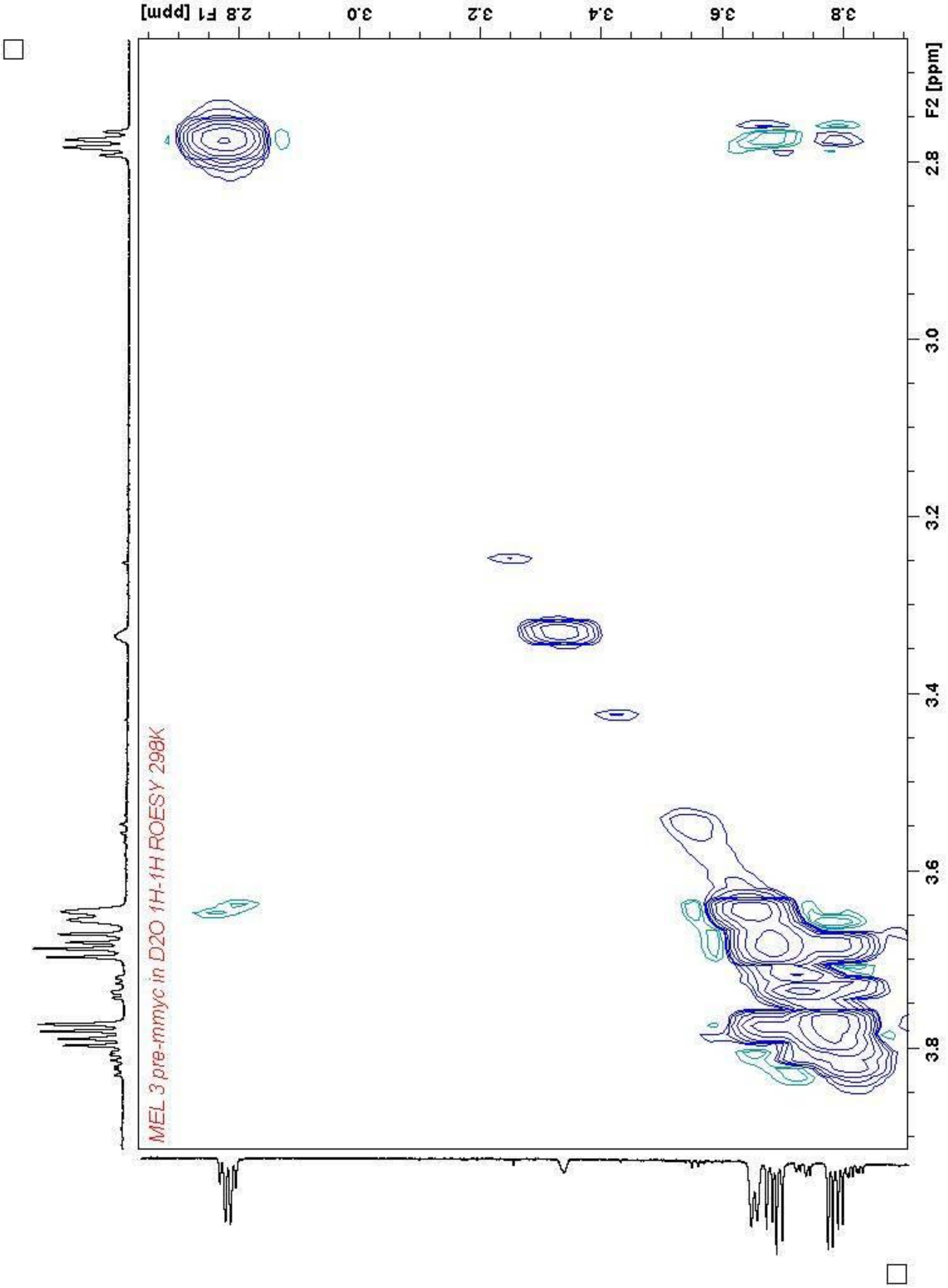

Figure S19. ROESY spectrum of pre-methylenomycin C (**6**) in D_2O

(A)

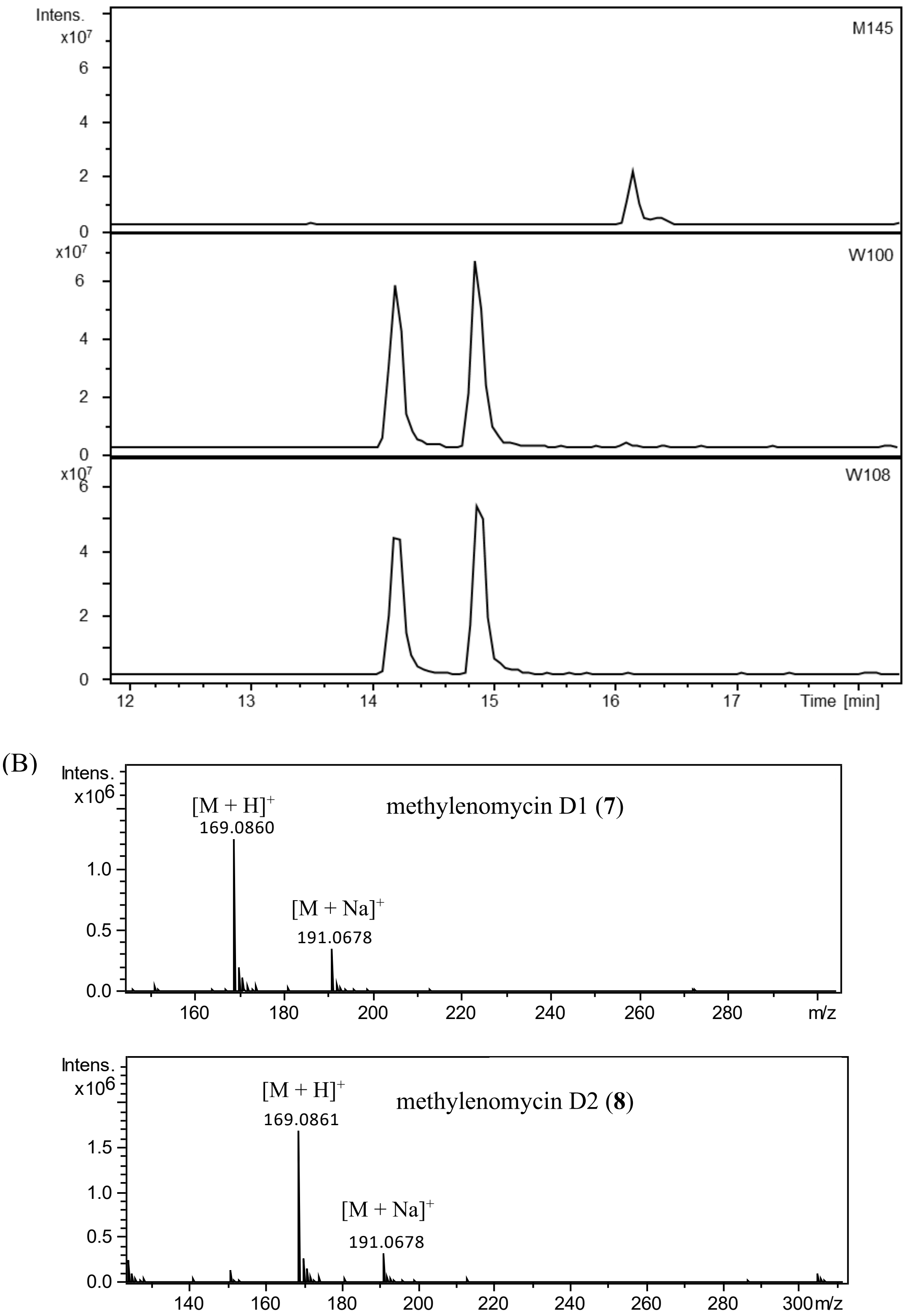

Figure S20. (A) Extracted ion chromatograms at *m/z* = 169.0860, corresponding to [M + H$^+$] for methylenomycin D1,**7** (14.2 min) and methylenomycin D2, **8** (14.9 min) from LC-MS analyses of extracts of *S. coelicolor* M145 (wild type), W100 (M145/C73_787/Δ*mmyF*/*mmyR*::*apr*), and W108 (M145/C73_787/Δ*mmyO*/*mmyR*::*apr*) grown for 3 days. (B) High-resolution mass spectra for **7** (top) and **8** (bottom), containing ions with *m/z* values corresponding to the [M + H$^+$] and [M + Na$^+$] ions.

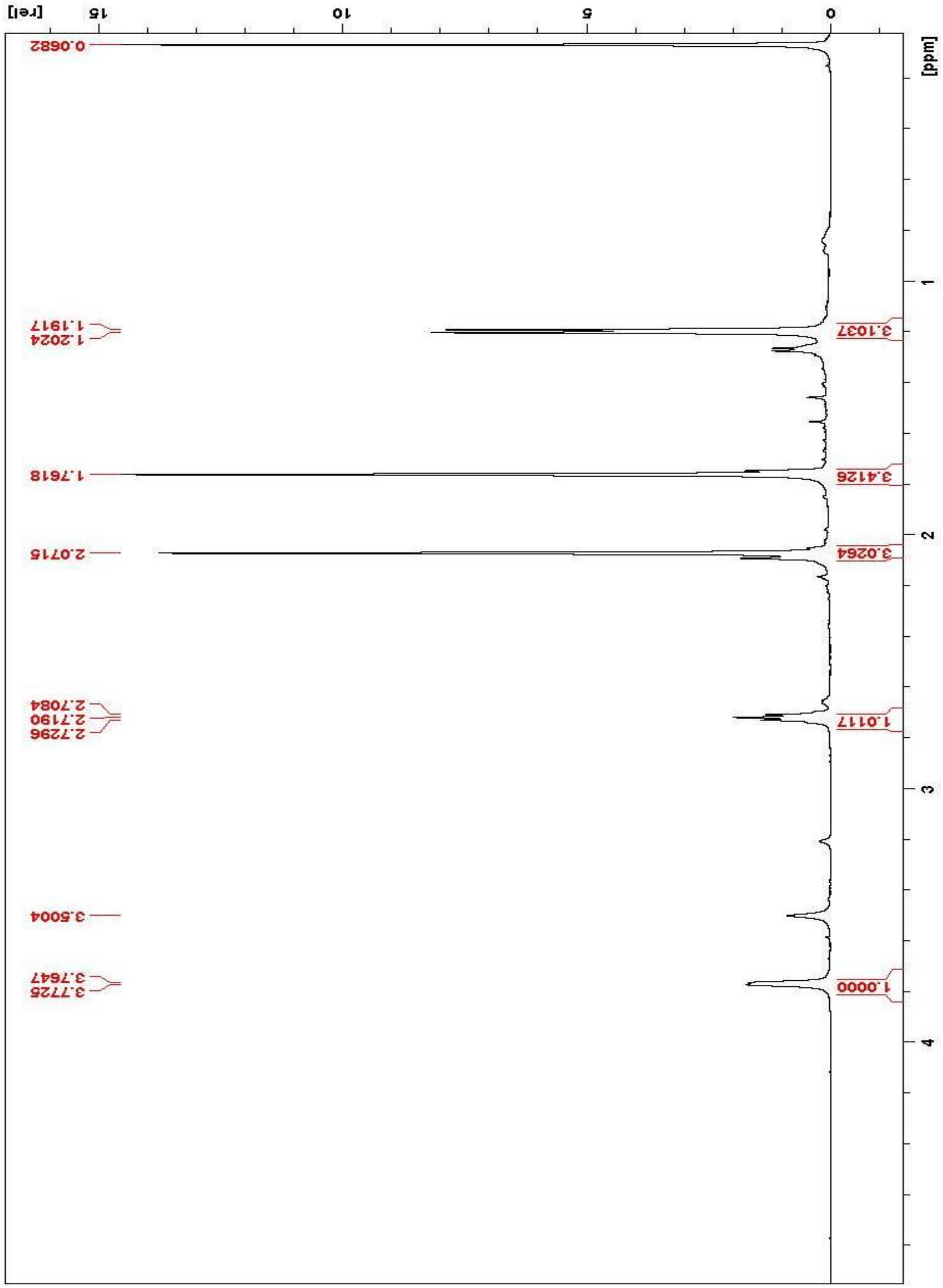

Figure S21. ^{1}H NMR spectrum of methylenomycin D1 (**7**) in $CDCl_3$

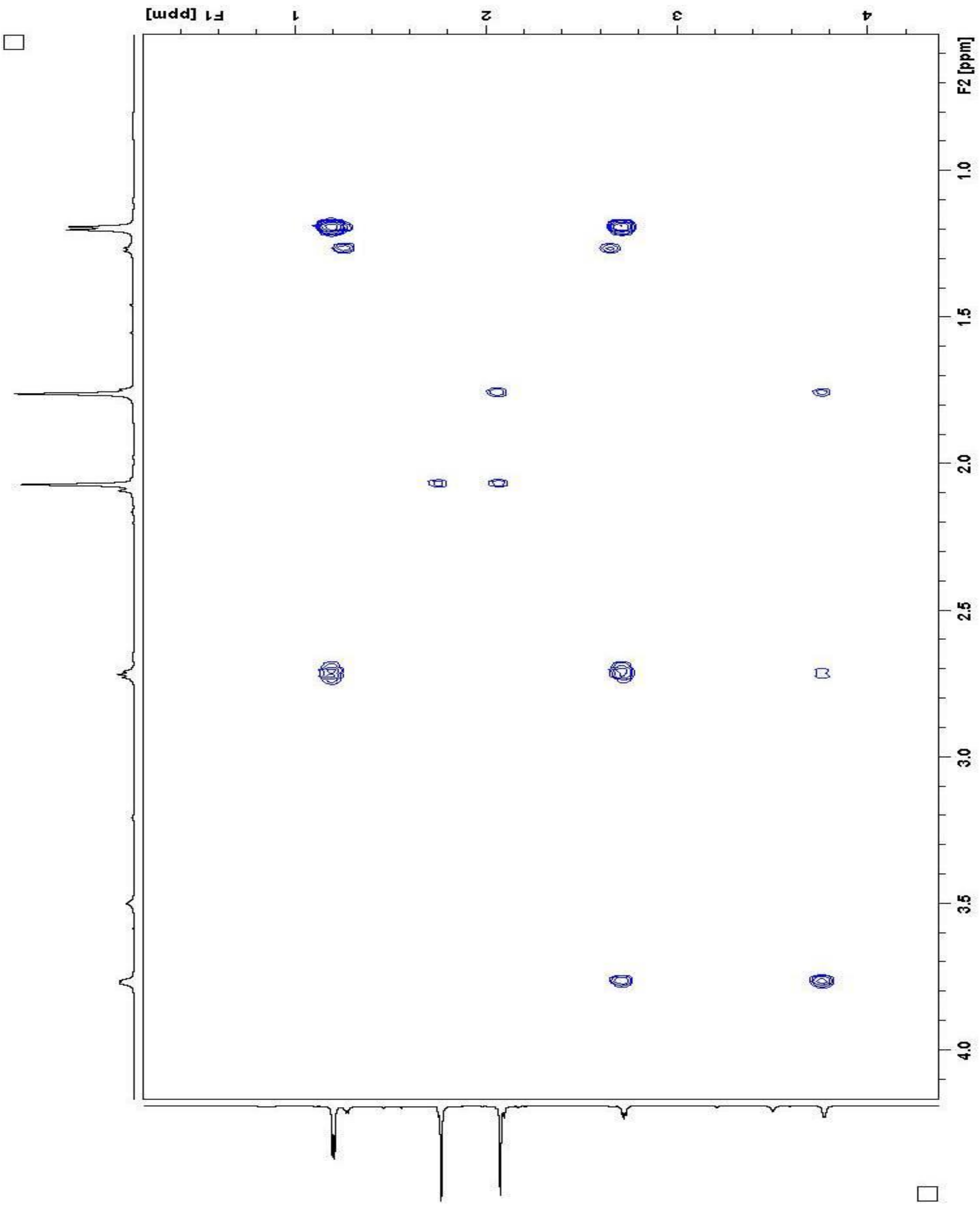

Figure S22. COSY spectrum of methylenomycin D1 (**7**) in $CDCl_3$

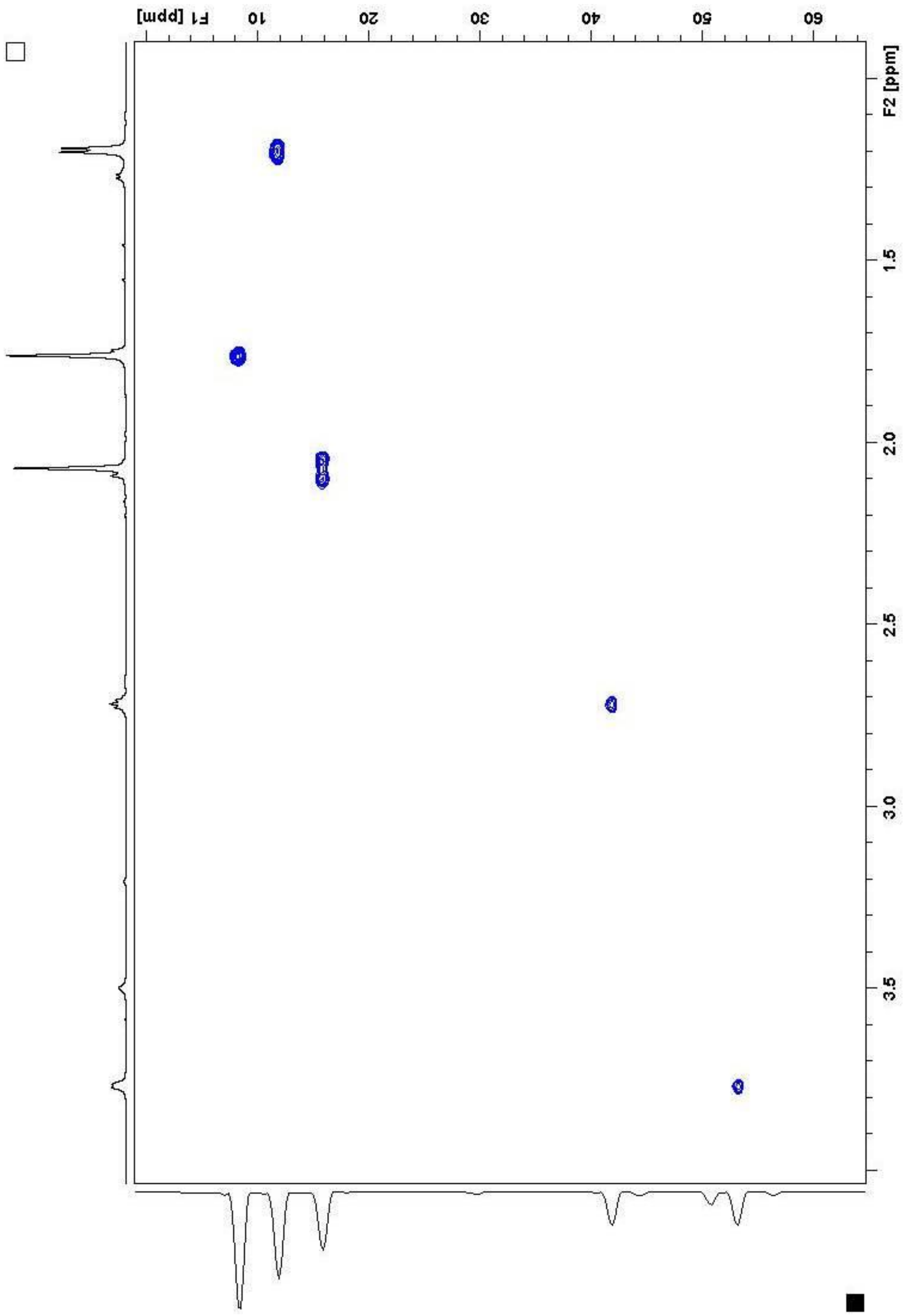

Figure S23. HSQC spectrum of methylenomycin D1 (**7**) in $CDCl_3$

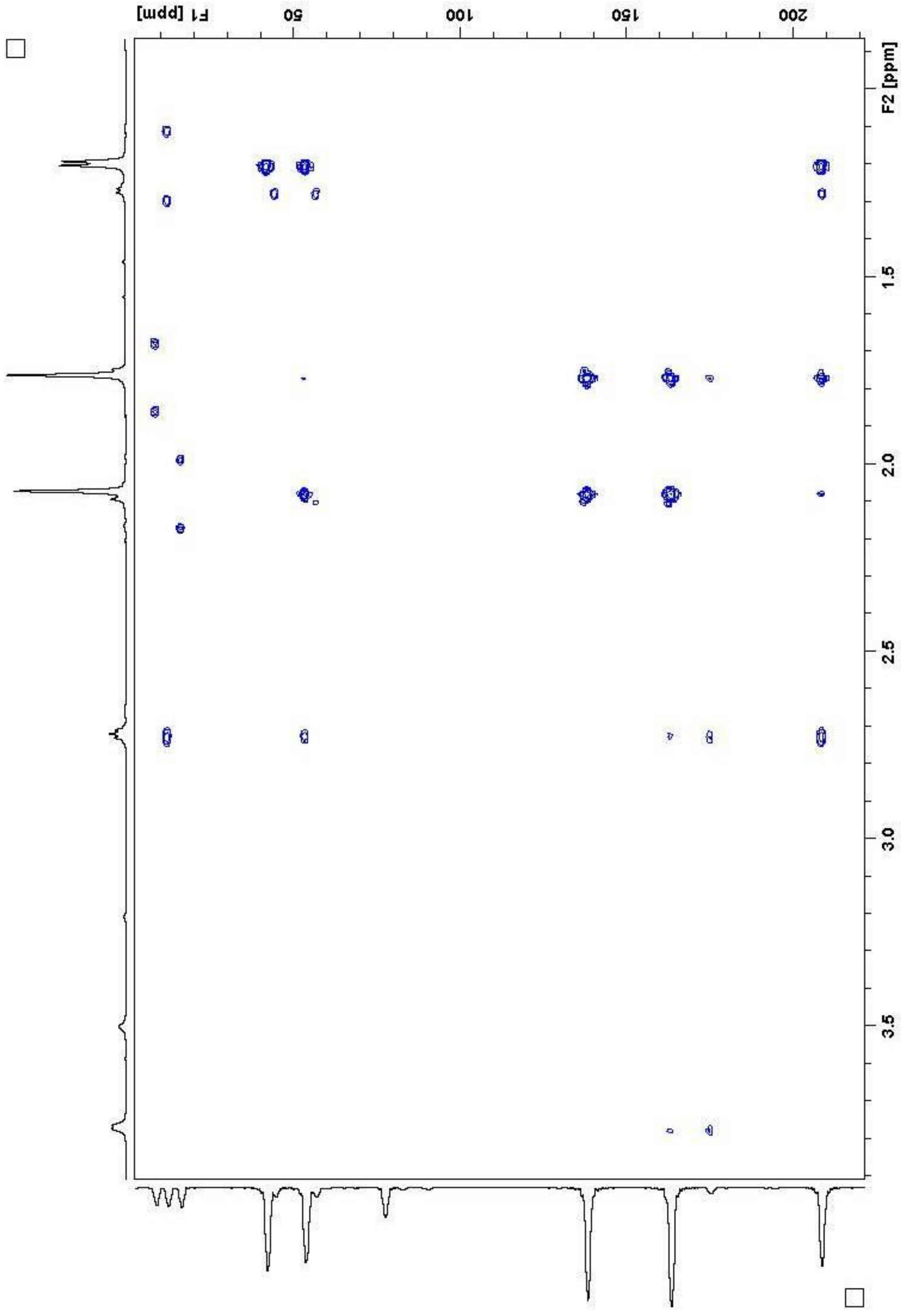

Figure S24. HMBC spectrum of methylenomycin D1 (**7**) in $CDCl_3$

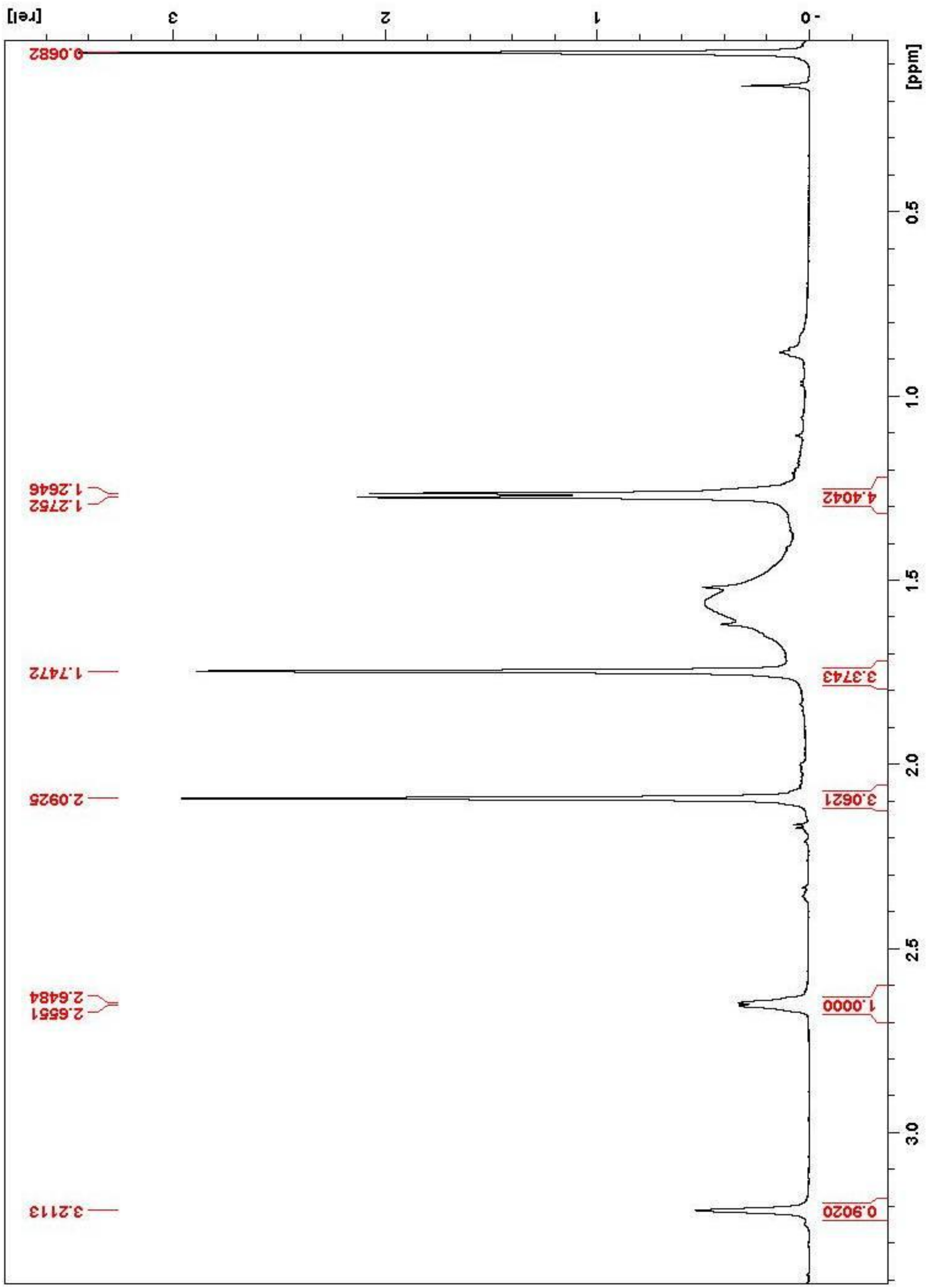

Figure S25. ^{1}H NMR spectrum of methylenomycin D2 (**8**) in $CDCl_3$

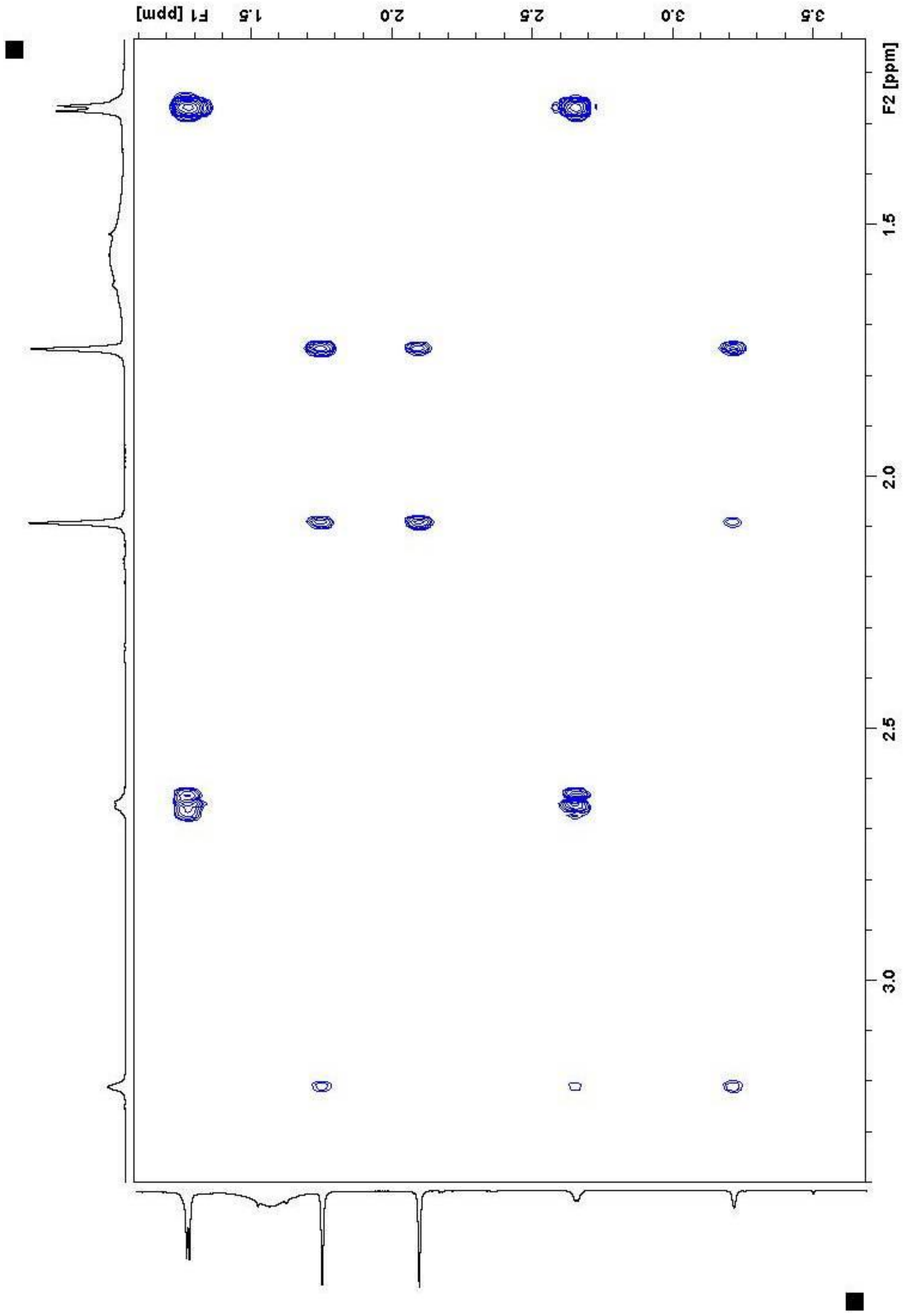

Figure S26. COSY spectrum of methylenomycin D2 (**8**) in $CDCl_3$

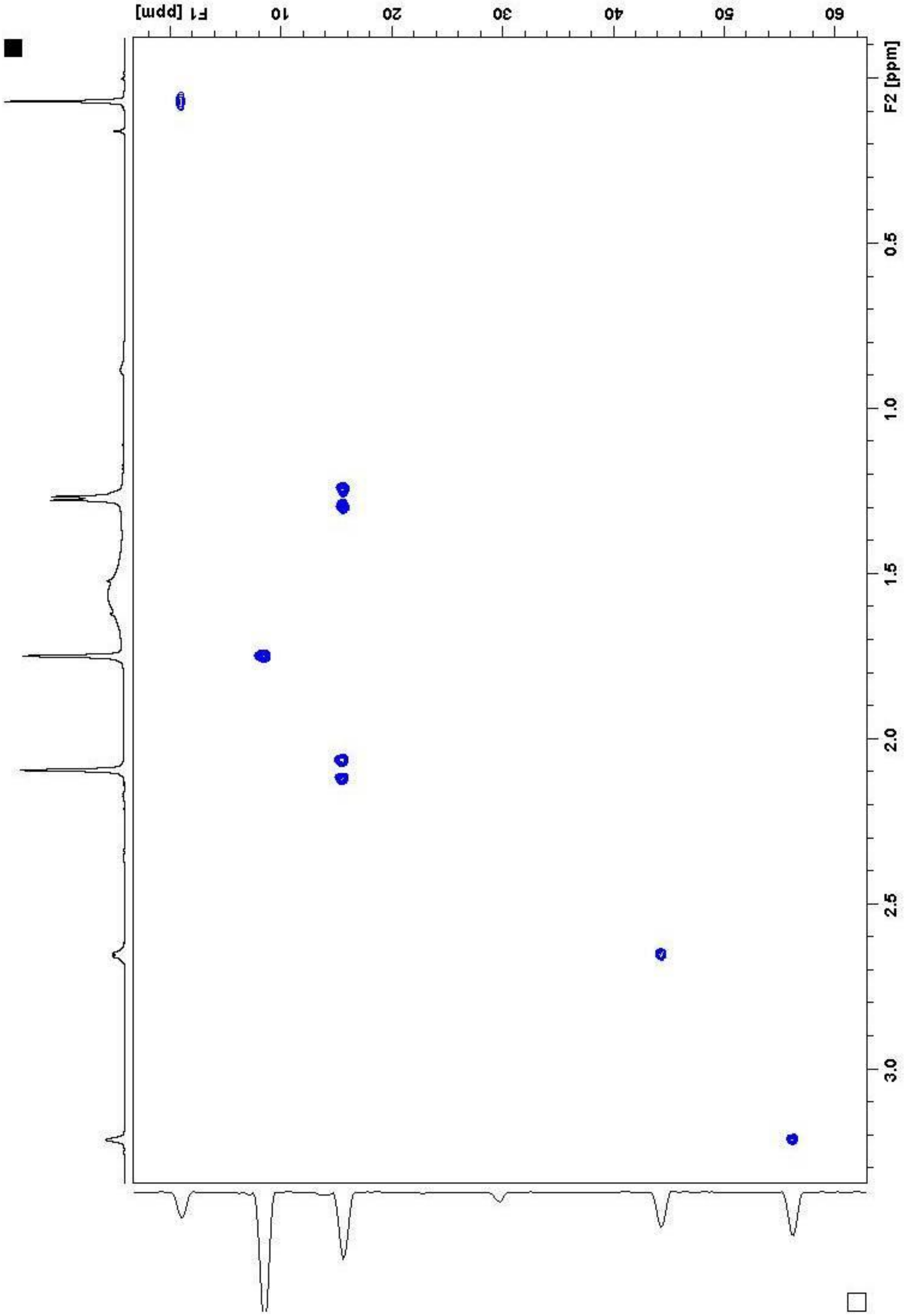

Figure S27. HSQC spectrum of methylenomycin D2 (**8**) in $CDCl_3$

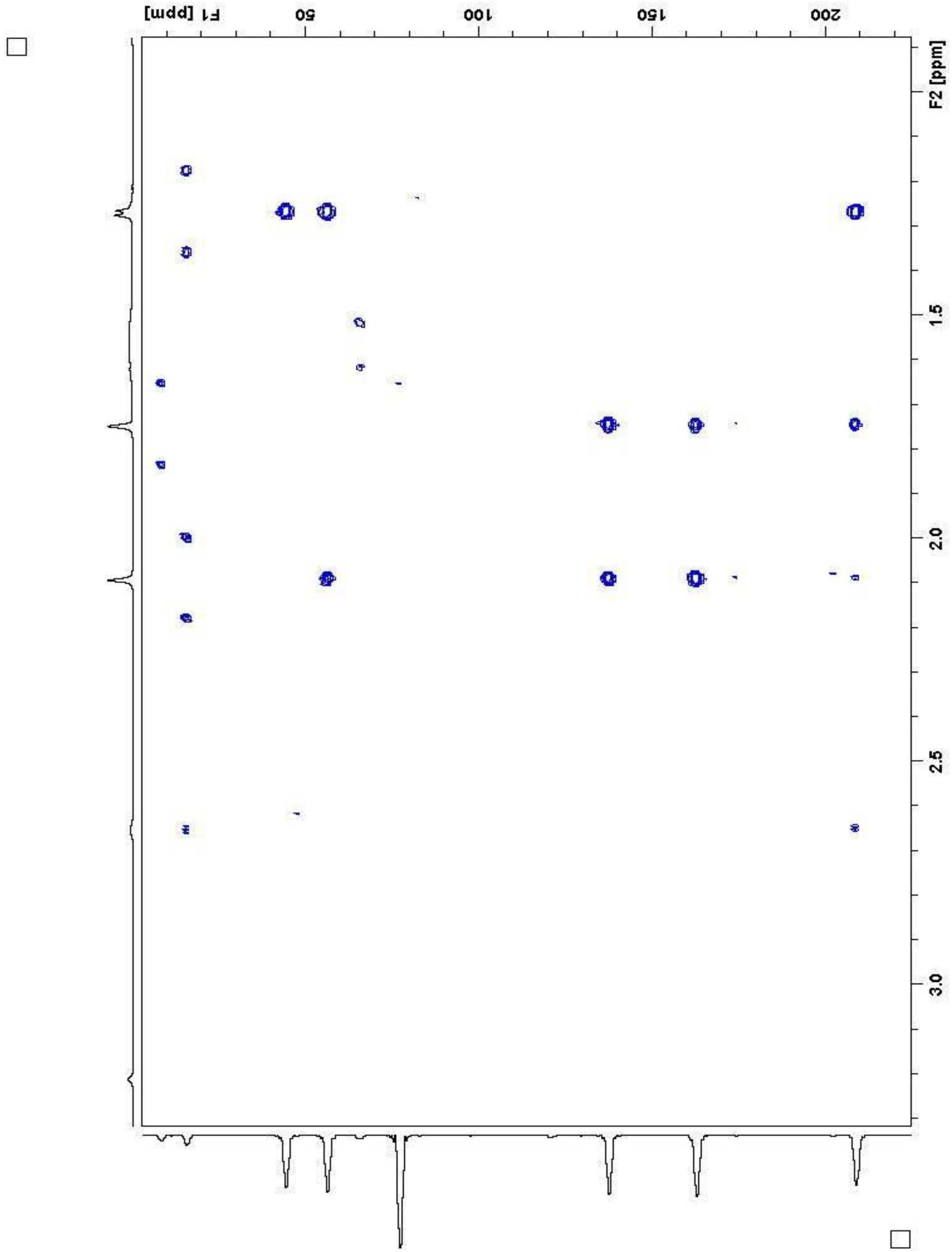

Figure S28. HMBC spectrum of methylenomycin D2 (**8**) in $CDCl_3$

Construction of plasmid pOSV556/mmyOF and introduction into* S. coelicolor *M145 to generate* S. coelicolor *W110.

The primers N/N'' and M''/M' (Table S4) were used to amplify the *mmyO* and *mmyF* genes using C73-787 as a DNA template. Following digestion using *Bam*HI, the *mmyO and mmyF* PCR products were isolated and ligated to generate a *mmyOF* DNA fragment that was isolated from the *mmyOO*, *mmyFF*, *mmyO* and *mmyF* fragment mixture by agarose gel electrophoresis and extraction. The purified *mmyOF* fragment was then digested using *Hin*dIII and *Xho*I and subcloned into the integrative plasmid pOSV556 to generate pOSV556/*mmyOF* (Fig. S29) The plasmid pOSV556/*mmyOF* carrying a hygromycin resistance marker was then introduced into *S. coelicolor* M145 *via* conjugation from *E. coli* ET12567/pUZ8002 to generate *S. coelicolor* W110 (Table S5).

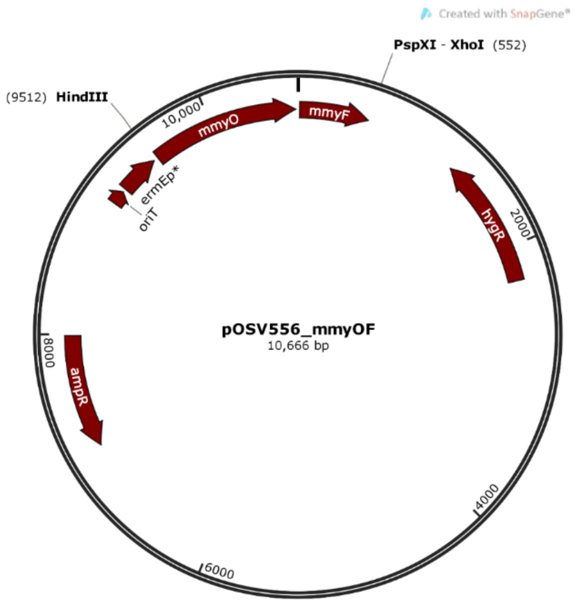

Figure S29. Map of pOSV556 carrying *mmyO* and *mmyF*.

Construction of plasmids pIJ86/mmr followed by introduction into* S. coelicolor *W110 and M145 to generate* S. coelicolor *W301 and W302, respectively.

The primers O/O' (Table S4) were used to amplify the methylenomycin resistance determinant gene *mmr* and its native ribosome binding site from C73-787. Following digestion using BamHI/HindIII, the *mmr* PCR product was isolated and subcloned in the replicative plasmid pIJ86 to generate pIJ86/*mmr* (Fig. S30). The plasmid pIJ86/*mmr* carrying an apramycin resistance marker was then introduced into *S. coelicolor* W110 and into M145 *via* conjugation from *E. coli* ET12567/pUZ8002 to generate *S. coelicolor* W301 and W302 (Table S5) respectively.

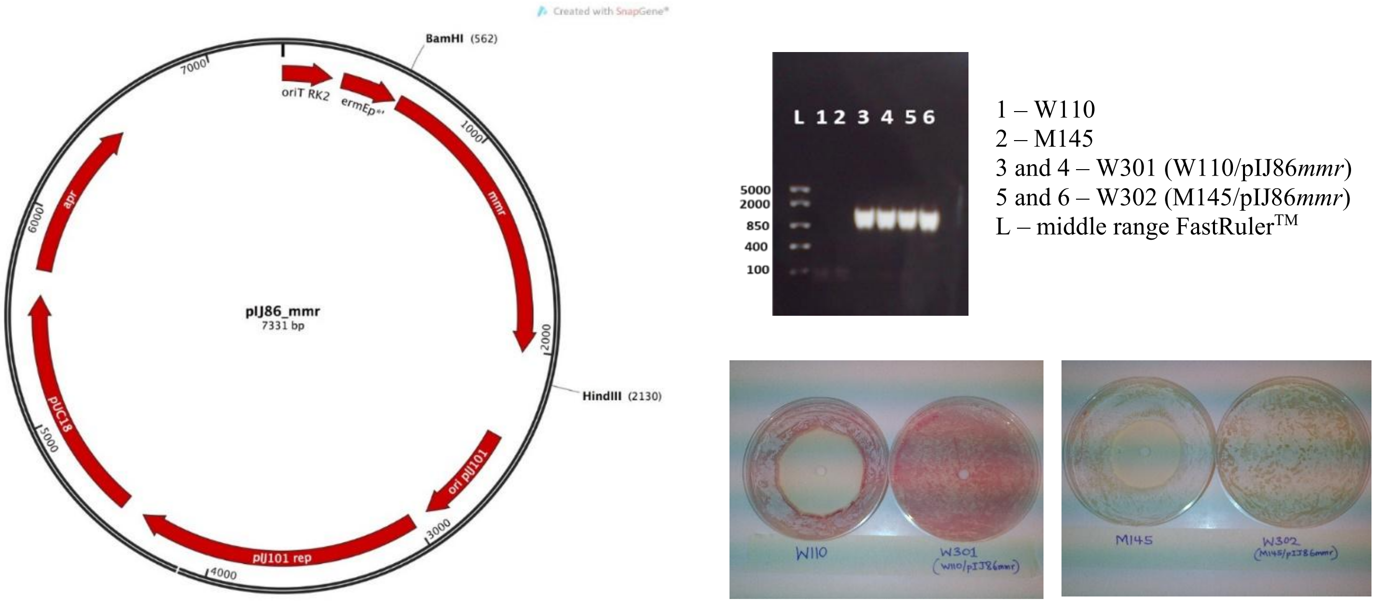

Figure S30. Map of pIJ86 carrying the methylenomycin resistance gene, *mmr* (Left). Construction of methylenomycin-resistant strains *of S. coelicolor* (Right): Top is colony PCR confirming presence of the 1.43kb *mmr* DNA in transconjugant strains W301 and W302, and absence from the parent strains W110 and M145, respectively. Filter paper discs impregnated with methylenomycin A caused a zone of inhibition on plates with the parent strains but not in their derivatives harbouring *mmr*.

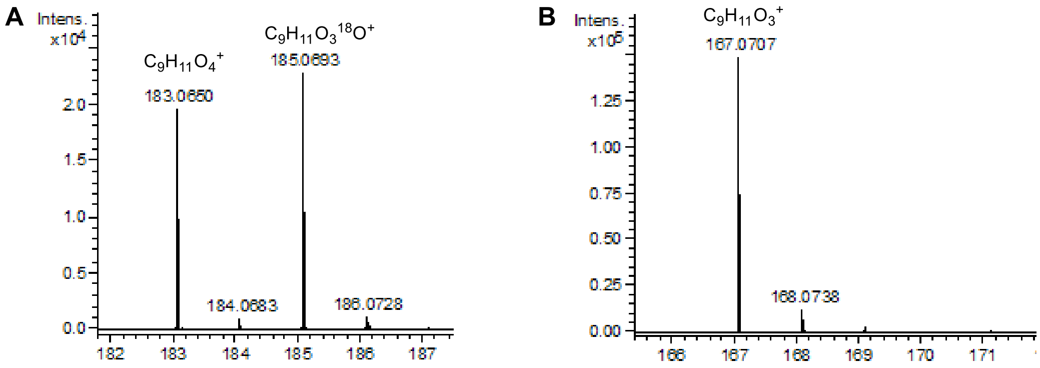

Figure S31. High resolution mass spectra of **1** and **2** from UHPLC-ESI-Q-ToF-MS analyses of organic extracts from *S. coelicolor* W89 grown under an $^{18}O_2$ atmosphere. (**A**) The spectrum from the peak corresponding to **1** revealed ions with *m/z* = 183.0650 and 185.0693, corresponding to $[M+H]^+$ for the unlabeled and singly 18O-labelled compound, respectively. (**B**) The spectrum from the peak corresponding to **2** revealed only an ion with *m/z* = 167.0707, corresponding to $[M+H]^+$ for the unlabeled species.

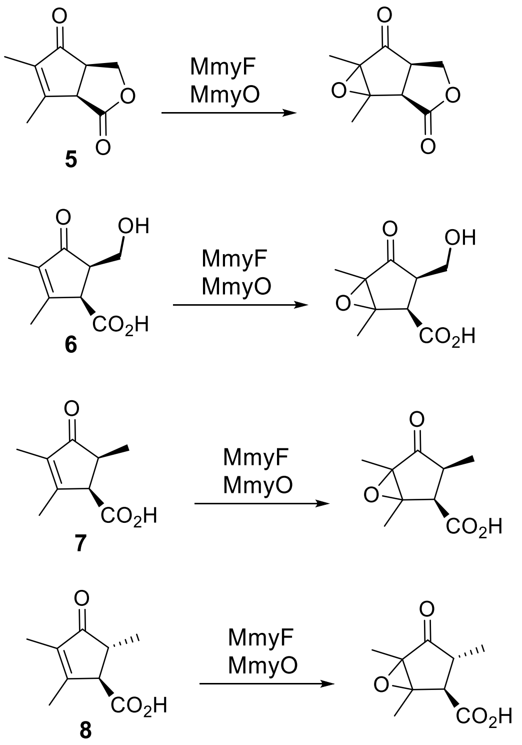

Figure S32. Possible products of epoxidation of pre-methylenomycin C lactone (**5**), pre-methylenomycin C (**6**), methylenomycin D1 (**7**) and methylenomycin D2 (**8**) by MmyF and MmyO.

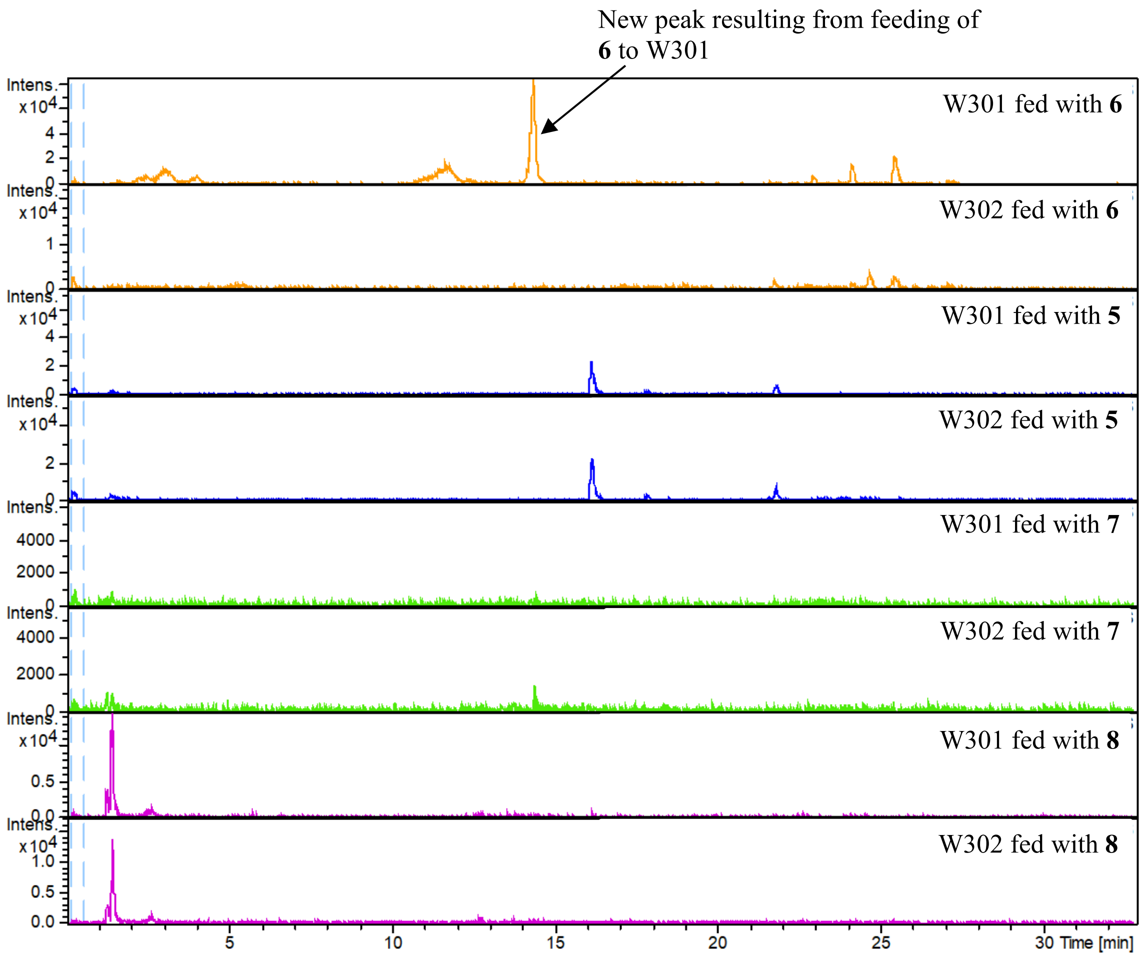

Figure S33. Extracted ion chromatograms (EICs) from LC-MS analyses of extracts of *S. coelicolor* W301 and W302 fed with **6**, **5**, **7** and **8**. Yellow: EIC at m/z = 201.07 and 223.07 corresponding to $[M + H]^+$ and $[M + Na]^+$ for possible epoxidized product of **6**. Blue: EIC at m/z = 183.06 and 205.06 corresponding to $[M + H]^+$ and $[M + Na]^+$ for possible epoxidized product of **5.** Green and Purple: EIC at m/z = 185.07 and 207.07 corresponding to $[M + H]^+$ and $[M + Na]^+$ for possible epoxidized products of **7** and **8** respectively**.**

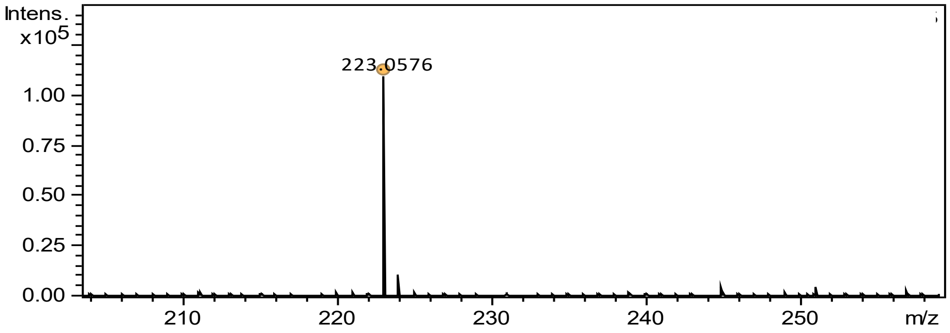

Figure S34. Mass spectrum of compound corresponding to the $[M + Na]^+$ ion for the epoxidized product (calculated m/z = 223.0577) resulting from the feeding of pre-methylenomycin C (**6**) to *S. coelicolor* W301.

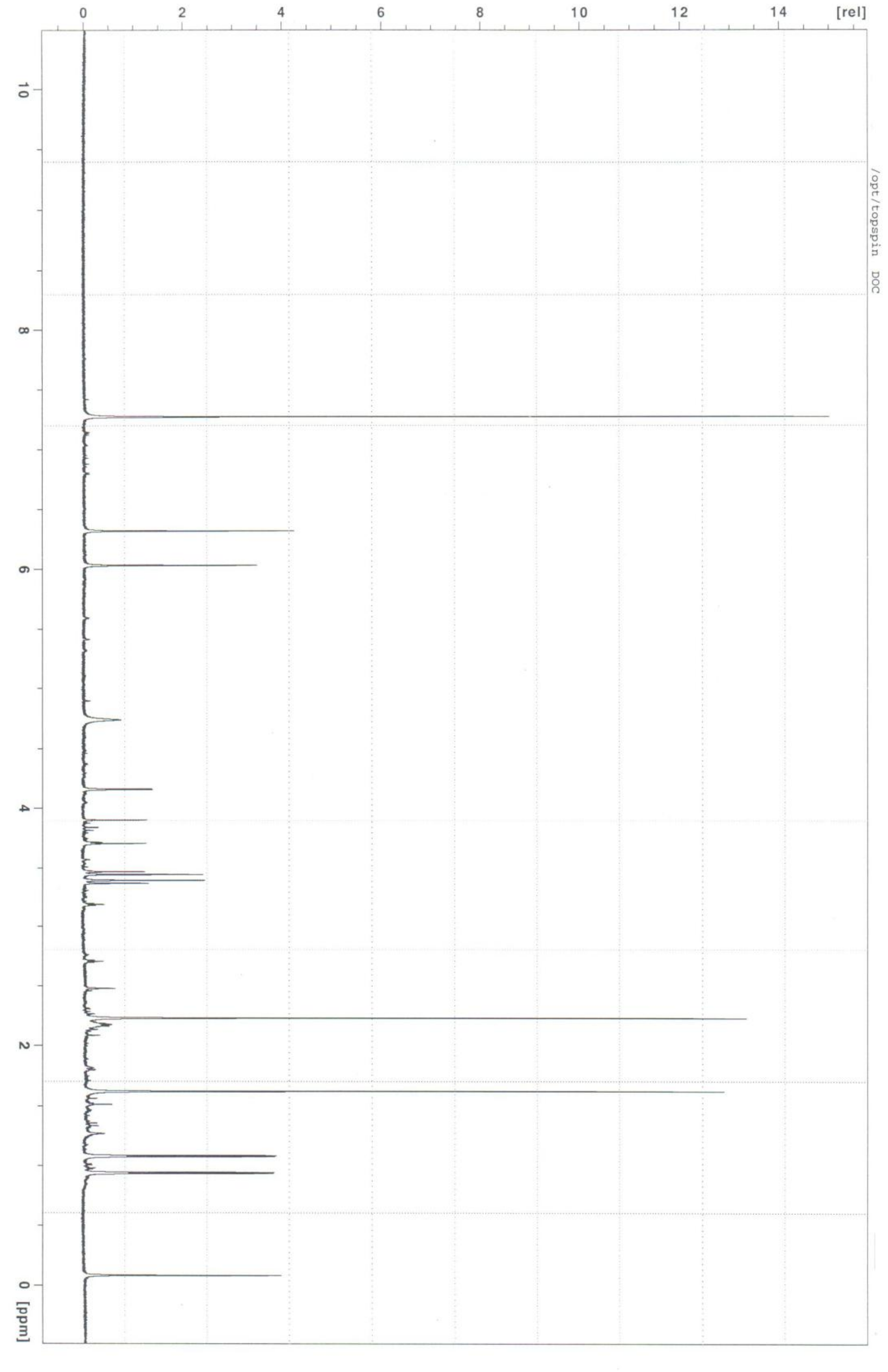

Figure S35. ^{1}H NMR spectrum of **10** in $CDCl_3$

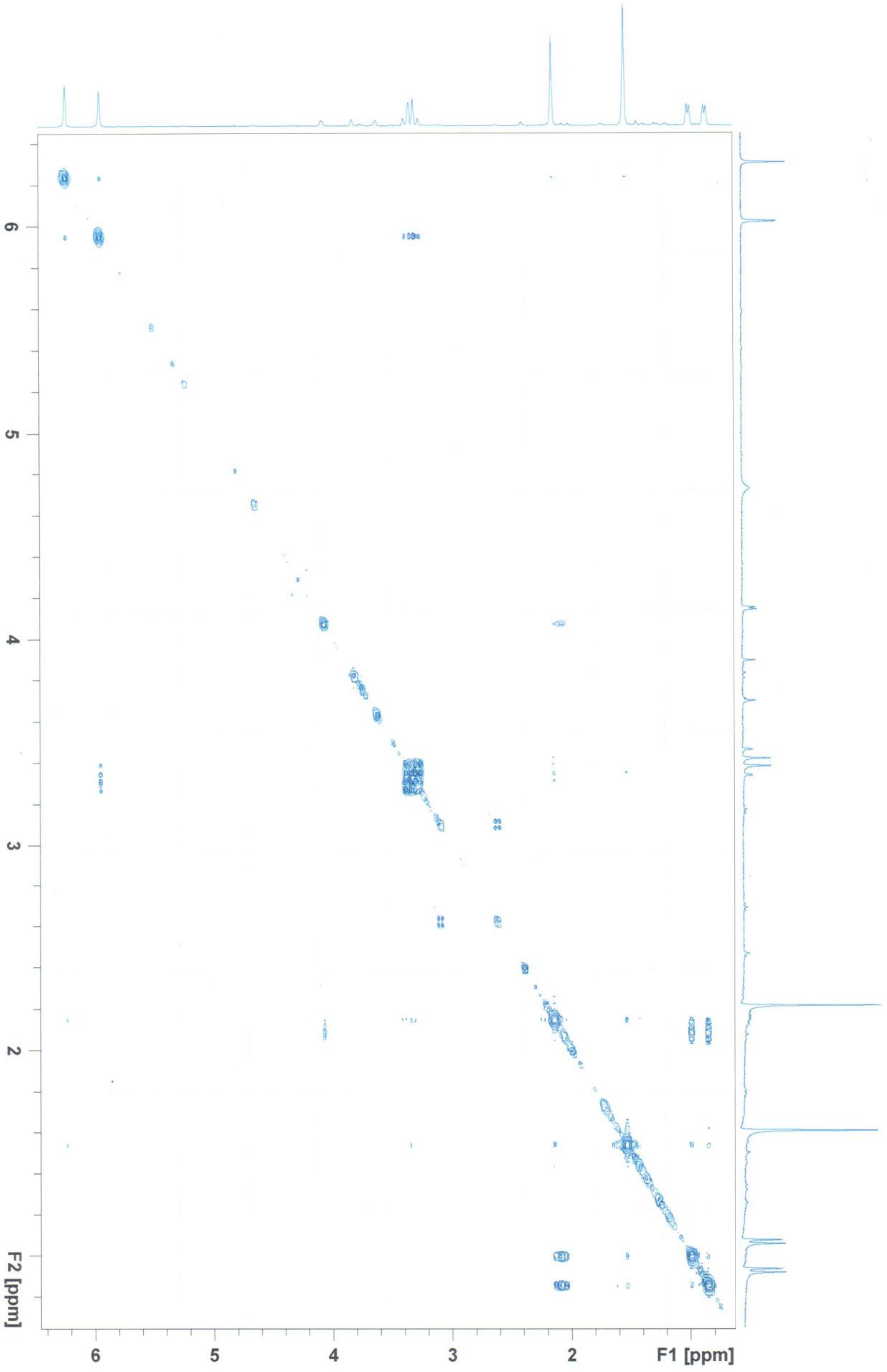

Figure S36. COSY spectrum of **10** in $CDCl_3$

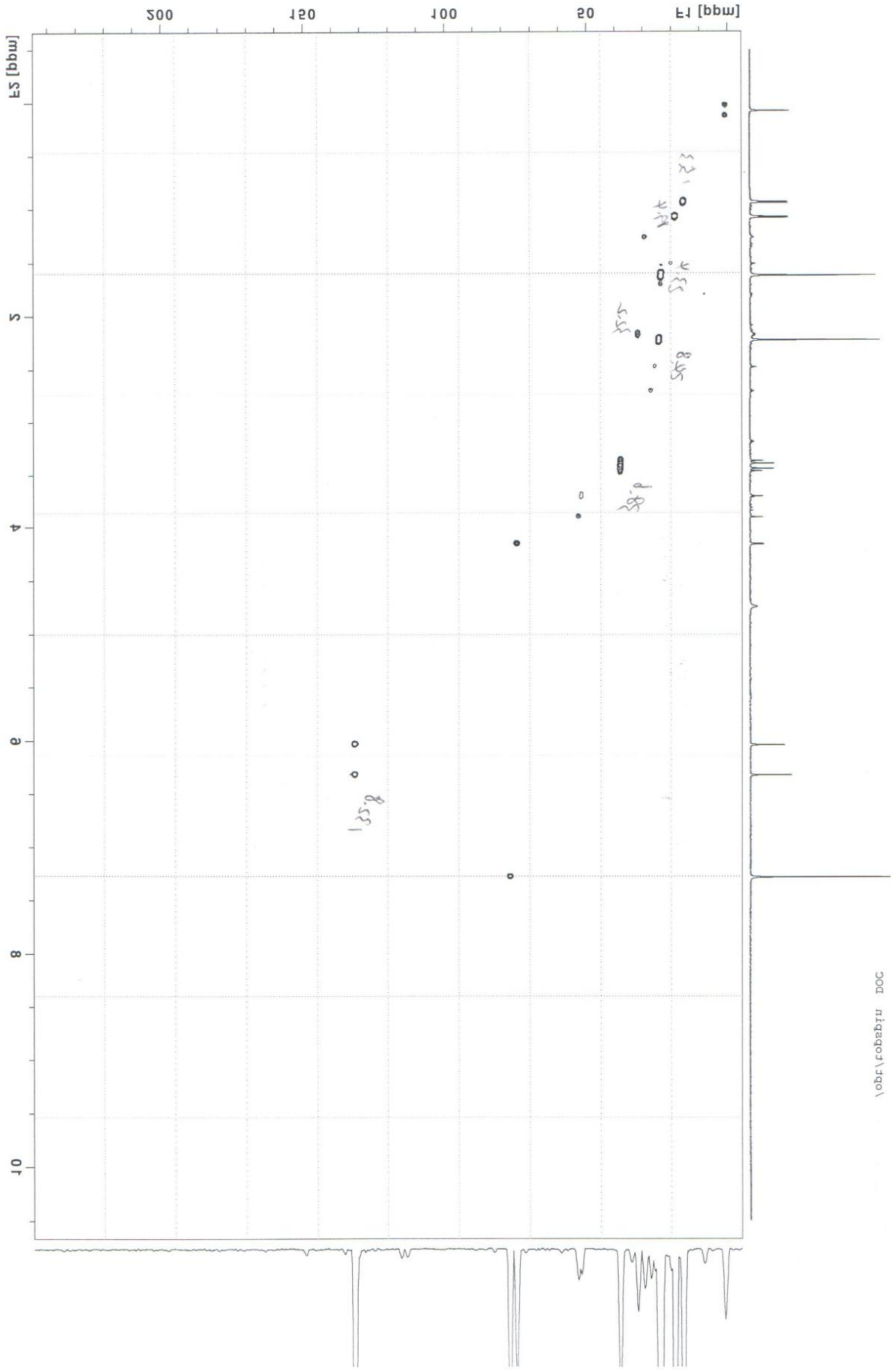

Figure S37. HSQC spectrum of **10** in $CDCl_3$

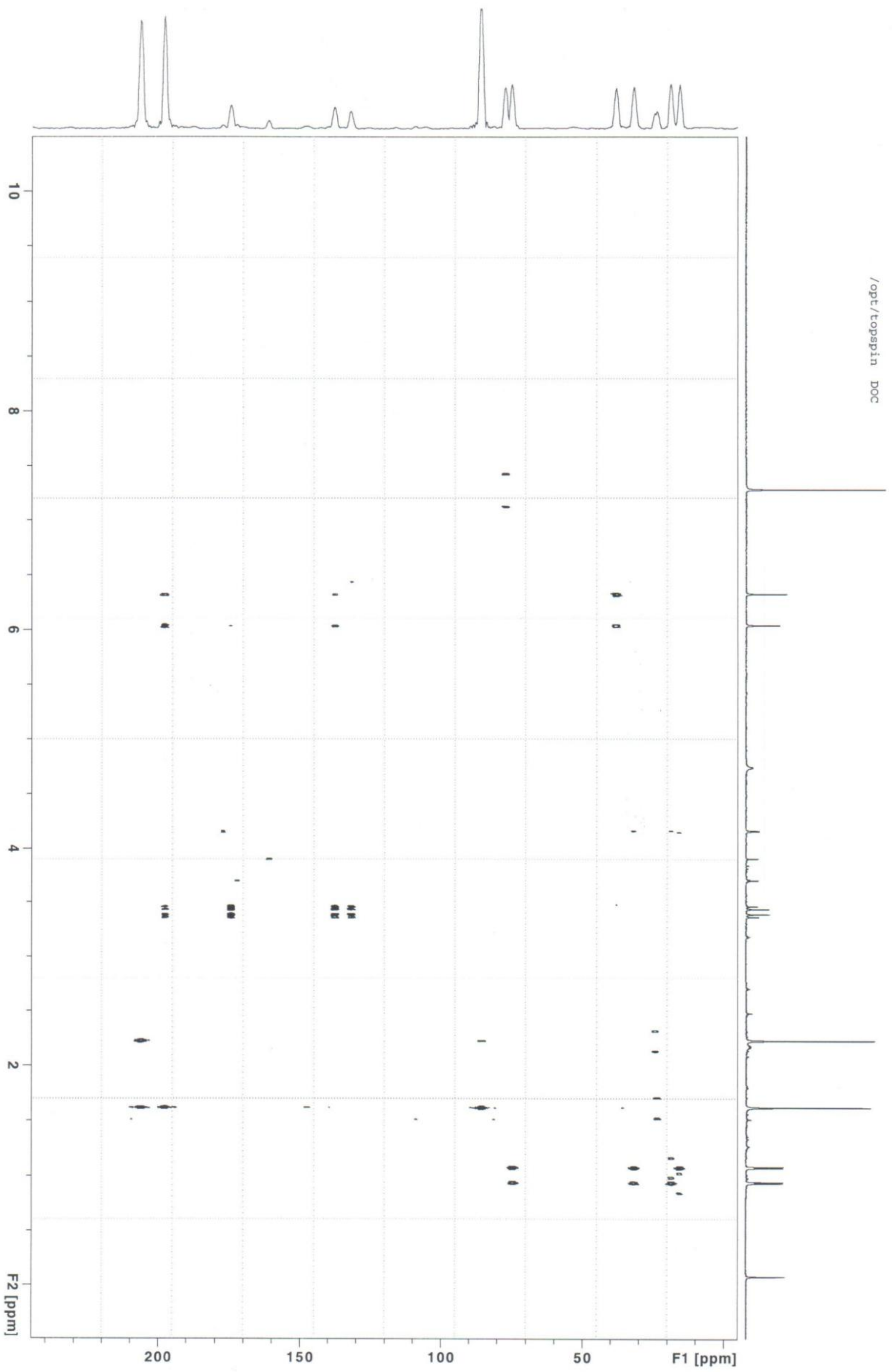

Figure S38. HMBC spectrum of **10** in $CDCl_3$

References

1. Takano, E.; Chakraburtty, R.; Nihira, T.; Yamada, Y.; Bibb, M.J. *Mol. Microbiol.* **2001**, *41*, 1015.

2. Corre, C.; Challis, G. L. *ChemBioChem.* **2005**, *6*, 2166.

3. CLSI. *Performance Standards for Antimicrobial Susceptibility Testing*, 25th Ed., Clinical Laboratory Standards Institute, 2015.

4. Ling, L. L.; Schneider, T.; Peoples, A. J.; Spoering, A. L.; En-gels, I., Conlon, B.P.; Mueller, A.; Schaberle, T. F.; Hughes, D.E.; Epstein, S.; Jones, M.; Lazarides, L.; Steadman, V. A.; Cohen, D. R.; Felix, C. R.; Fetterman, K. A.; Millett, W. P.; Nitti, A. G.; Zullo, A. M.; Chen, C.; Lewis, K. *Nature* **2015**, *517*, 455.

5. Shiloh, M.U.; Ruan, J.; Nathan, C. *Infect. Immun.* **1997**, *65*, 3193.

6. Hartkoorn, R. C.; Uplekar, S.; Cole, S. T. *Antimicrob. Agents Chemother.* **2014**, *58*, 2979.

7. Gust, B.; Challis, G. L.; Fowler, K.; Chater, K. F. *Proc. Natl. Acad. Sci. USA* **2003**, 100, 1541.

8. Gust, B.; Chandra, G.; Jakimowicz, D.; Yuqing, T.; Bruton, C. J.; Chater, K. F. *Adv. Appl. Microbiol.* **2004**, 54,107.

9. Haynes, S.W.; Sydor, P.K.; Stanley, A.E.; Song, L.; Challis, G.L. *Chem. Commun.* **2008**, 1865.

10. Kieser, T.; Bibb, M. J.; Buttner, M. J.; Chater, K. F.; Hopwood, D. A. *Practical Streptomyces Genetics*, The John Innes Foundation, Norwich, 2000.
